# Engineering the magnetic coupling and anisotropy at the molecule–magnetic surface interface in molecular spintronic devices

**DOI:** 10.1038/ncomms13646

**Published:** 2016-12-08

**Authors:** Victoria E. Campbell, Monica Tonelli, Irene Cimatti, Jean-Baptiste Moussy, Ludovic Tortech, Yannick J. Dappe, Eric Rivière, Régis Guillot, Sophie Delprat, Richard Mattana, Pierre Seneor, Philippe Ohresser, Fadi Choueikani, Edwige Otero, Florian Koprowiak, Vijay Gopal Chilkuri, Nicolas Suaud, Nathalie Guihéry, Anouk Galtayries, Frederic Miserque, Marie-Anne Arrio, Philippe Sainctavit, Talal Mallah

**Affiliations:** 1 Institut de Chimie Moléculaire et des Matériaux d’Orsay (ICMMO), CNRS, Université Paris Sud, Université Paris Saclay, 91405 Orsay, France; 2 SPEC, CEA, CNRS, Univesité Paris Saclay, CEA Saclay, 91191 Gif-sur-Yvette, France; 3 IPCM, UMR CNRS 7201, UPMC, Université Pierre et Marie Curie, F-75005 Paris, France; 4 Unité Mixte de Physique CNRS/Thales, 1 Avenue Auguste Fresnel, 91767 Palaiseau, France and Université Paris-Sud, 91405 Orsay, France; 5 Synchrotron SOLEIL, L’Orme des Merisiers Saint-Aubin—BP 48, 91192 Gif-sur-Yvette, France; 6 Laboratoire de Chimie et Physique Quantiques, Université de Toulouse III, 118, route de Narbonne, 31062 Toulouse, France; 7 PSL Research University, Chimie ParisTech-CNRS, Institut de Recherche de Chimie Paris, F-75005 Paris, France; 8 CEA/DEN/DANS/DPC/SCCME, Laboratoire d'Etude de la Corrosion Aqueuse, F-91191 Gif-sur-Yvette, France; 9 IMPMC-CNRS, Université Pierre et Marie Curie, F-75005 Paris, France

## Abstract

A challenge in molecular spintronics is to control the magnetic coupling between magnetic molecules and magnetic electrodes to build efficient devices. Here we show that the nature of the magnetic ion of anchored metal complexes highly impacts the exchange coupling of the molecules with magnetic substrates. Surface anchoring alters the magnetic anisotropy of the cobalt(II)-containing complex (Co(Pyipa)_2_), and results in blocking of its magnetization due to the presence of a magnetic hysteresis loop. In contrast, no hysteresis loop is observed in the isostructural nickel(II)-containing complex (Ni(Pyipa)_2_). Through XMCD experiments and theoretical calculations we find that Co(Pyipa)_2_ is strongly ferromagnetically coupled to the surface, while Ni(Pyipa)_2_ is either not coupled or weakly antiferromagnetically coupled to the substrate. These results highlight the importance of the synergistic effect that the electronic structure of a metal ion and the organic ligands has on the exchange interaction and anisotropy occurring at the molecule–electrode interface.

## Abstract (web summary)

Controlling the magnetic response of a molecular device is important for spintronic applications. Here the authors report the self-assembly, magnetic coupling, and anisotropy of two transition metal complexes bound to a ferrimagnetic surface, and probe the role of the nature of the transition metal ion.

With the quest to design electronic devices ever smaller, molecules have gained a place in the realm of not only traditional electronics, but also in spintronics. Molecular spintronics is a multidisciplinary field of research that unites the exceptional properties of molecules with the requirements of spin-based technology^1^,^2^. It has been shown that the magneto-resistance response of a molecular spintronic device hinges upon the interface between the ferromagnetic surface and the molecular layer^3^. Mastering the quality and the nature of this interface is of paramount importance for the construction of devices with reproducible responses^3^. A major challenge is to build devices with defined chemical bonding between the magnetic molecules and the magnetic electrodes allowing a control over the nature of the exchange coupling interaction and thus over the magneto-resistance response of the device^4^. Single magnetic molecules with designed geometry, architecture and electronic structure allow scientist to attain this goal^5^,^6^.

Recent endeavours towards molecular spintronics include (but are not limited to) the following examples. Mannini and co-workers demonstrated that the magnetic memory effect is retained at low temperature (<1.0 K) when single molecule magnets (SMMs) are wired not only onto non-magnetic gold surfaces^7^,^8^ but also onto magnetic surfaces commonly used as ferromagnetic electrodes in spintronic devices, such as the oxide lanthanum strontium manganite (LSMO) and metallic cobalt^9^. When ferromagnetic surfaces were employed, terbium(III) double-decker (TbPc_2_) molecules organized with different orientations (parallel or perpendicular) depending on the surface employed and did not show significant magnetic interaction between the molecules and the substrate^9^. In alternative study, Rizzini *et al*.^10^ demonstrated that it is possible to induce exchange bias in a small fraction of the TbPc_2_ molecules adsorbed onto a manganese surface. Another class of materials, paramagnetic porphyrin molecules (Mn-porphyrin and Fe-porphyrin) and phthalocyanine, are known to physically adsorb onto ferromagnetic Ni or Co surfaces and show surface induced magnetic ordering and hysteresis^5^,^11^,^12^,^13^,^14^,^15^,^16^.

Although these systems are promising, interface engineering is necessary to fully control and comprehend the nature of the interaction between the magnetic molecular layer and the ferromagnetic surface. To do so, we design metal-containing (M= Ni(II) and Co(II)) magnetic complexes that can chemically bond to epitaxial Fe_3_O_4_ (111), which is chosen as the magnetic substrate, thus defining the interface between the molecules and the electrodes. This step allows us performing the analysis of the exchange coupling interaction responsible for the magneto-resistance response. Phosphonates (R-PO(OH)_2_) are known to react with a variety of metal-oxides and form stable monolayers through a heterocondensation reaction that results in strong *Fe-O-P*(O(OH))-R bonds and the release of water molecules^17^. Taking into account these design criteria, we prepare, on one hand, metal complexes bearing phosphonate groups able to make coordination bonds with a metal-oxide surface, and on the other hand, we choose the Fe_3_O_4_ (111) epitaxial metal oxide surface as the magnetic substrate in order not only to ensure stable coordination bond with the designed complex but also to privilege as much as possible its orientation. Surface anchoring induces, in the paramagnetic cobalt(II)-containing complex, a magnetic hysteresis at the Co-edge evidenced by X-ray magnetic circular dichroism (XMCD) studies and indicative of magnetic coupling with the magnetic surface. We do not observe hysteresis at the Ni-edge for the isostructural nickel(II)-containing complex consistent with the absence of magnetic interaction with the surface. First principle *ab initio* calculations indicate that the Co(II) complex is relatively strongly ferromagnetically coupled with the surface, while the Ni(II) one undergoes a much weaker exchange coupling that is antiferromagnetic. These results highlight the fundamental effect that the electronic structure of a metal ion in conjunction with the nature of the organic spacer has on the resulting molecule/magnetic surface interaction.

## Results

### Design and synthesis of the molecules

When designing a versatile system that could be exploited in molecular spintronic applications such as spin filtering or spin transfer torque, we took into consideration two main factors. First, we wanted to construct a well-defined molecules/substrate interface by using anchoring groups as spacers that would graft reproducibly and in a controlled manner onto the oxide of choice. This feature, in principle, will allow engineering the molecule/magnetic surface magnetic and electronic interactions. Second, these complexes must be air stable and more importantly thermodynamically stable in solution to prevent possible reactions between the metal ion, belonging to the complex, and the surface.

Many different anchoring groups are known to graft to oxide surfaces such as phosphonates, carboxylates and methyl silanes^18^,^19^. Amongst them all, the phosphonic acid moiety was chosen because it is known to covalently tether to a variety of oxides (for example TiO_2_, Ta_2_O_5_, LSMO, ZnO and Fe_3_O_4_)^20^,^21^,^22^, is stable in a variety of solvents, in a large range of pH, and is insensitive to hydrolysis^17^,^23^,^24^.

**Figure 1:**
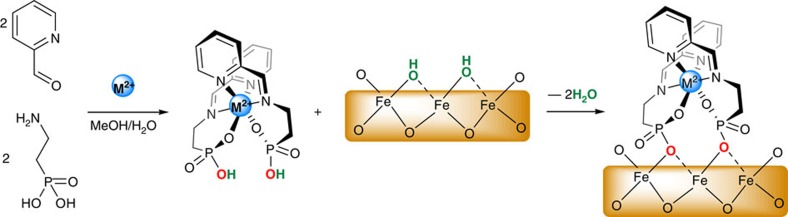
Synthesis of the complexes and surface deposition. Schematic view of the formation of the metal ion containing complexes (M=Co and Ni) and their anchoring to the metal iron-oxide substrate by the formation of M-OPO-Fe bonds and the releasing of water molecules.

The organic ligand and the related complexes were designed with three criteria in mind: (i) maximum thermodynamic stability, (ii) facile modification of the nature of the magnetic ion keeping everything else unchanged, and (iii) optimal orientation of the phosphonic acid groups that play the role of spacer units between the magnetic ions and the substrate. This last criterion enables the electronic communication between the surface and the magnetic complexes, and directly impacts the nature and the magnitude of their exchange coupling. The one pot reaction between 2-(aminoethyl)phosphonic acid (2 equiv.), 2-pyridinecarboxaldehyde (2 equiv.) and the corresponding hydrated M^II^(CH_3_COO)_2_ salt (1 equiv.), using the metal ion M^II^(Co^II^ or Ni^II^) as a template, produced bisimine complexes Co- and Ni(Pyipa)_2_ as the uniquely observed products (Fig. 1, the tridentate ligand Pyridin-2-ylmetylene)amino]ethyl phosphonic acid (Pyipa) is produced *in situ*, for full characterization of the complexes see Methods, and Supplementary Discussion).

It is important to notice (Fig. 1) that the organic ligand Pyipa was designed to be tridendate ensuring a large thermodynamic (entropic) stability of the M(Pyipa)_2_ complexes. Furthermore, the assembly of two Pyipa ligands by the metal ions leads to only one possible isomer with the phosphonate groups pointing in the same direction (Fig. 1). The two OH species belonging to the two phosphonate groups are expected to play the role of a ‘chelate’ towards the Fe_3_O_4_ (111) epitaxial surface, thus bringing an additional (entropic) thermodynamic stabilization to the formation of the complexes/substrate coordination bonds. And finally, the bulkiness of the complex is expected to preclude its large tilting when anchored to the surface, keeping its C_2_ symmetry axis very close to perpendicular to the substrate.

**Figure 2:**
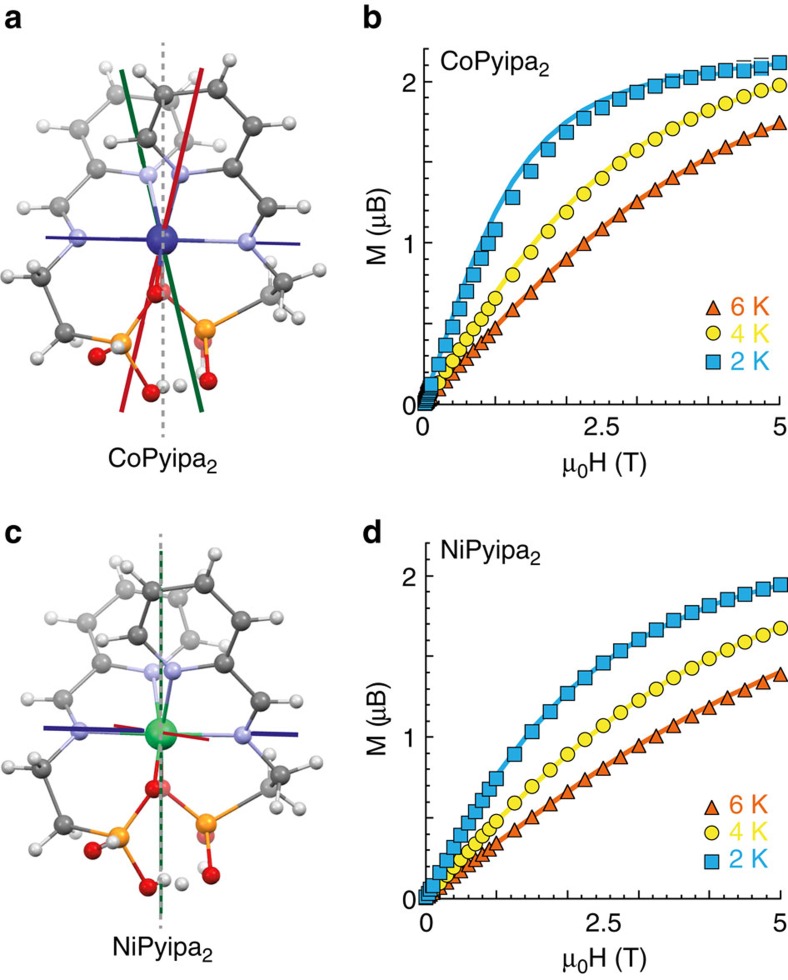
Complex Co- and Ni(Pyipa)_2_. X-ray crystal structure of Co(Pyipa)_2_ (**a**) and Ni(Pyipa)_2_ (**c**); C, grey; N, lilac; O, red; P, orange; H, white; Co, blue; Ni, green. The red, green and blue axes represent the *x*, *y*, *z* direction of the anisotropy tensor, respectively. The magnetization as a function of field plots for Co(Pyipa)_2_ (**b**) and Ni(Pyipa)_2_ (**d**) solid lines correspond to the best fits; see SI for the parameter values.

Single crystal X-ray analysis revealed that Co- and Ni(Pyipa)_2_ are isostructural (Fig. 2; Supplementary Table 1; Supplementary Data 1-2). In both complexes the metal centre is bound to the two tridentate ligands, leading to a distorted octahedral environment and molecular C_2v_ symmetry. One oxygen atom of each phosphonate group is coordinated to the metal(II) ion leaving the other oxygen atoms available to react with the iron oxide surface. It is worth noting that (i) the distance between two oxygen atoms belonging to the two Pyipa ligands is equal to 2.59 Å very close to the distance between two oxygen atoms belonging to the Fe_3_O_4_ (111) surface (2.85 Å) ensuring little deformation of the coordination sphere of the metal ions upon the grafting of the complexes onto the substrate.

The magnetic behaviour of the complexes was studied by direct current (d.c.) magnetic susceptibility and magnetization measurements (Fig. 1; Supplementary Figs 1 and 2) and by *ab initio* calculations that used the spin-orbit state interaction method (Supplementary Discussion: ‘*Ab initio* calculations of the complexes’)^25^,^26^,^27^. The calculations and the experimental data are in good agreement and indicate the presence of an easy plane of magnetization for Co(Pyipa)_2_ and an easy axis for Ni(Pyipa)_2_ (Table 1). We extracted the orientation of the magnetization axis from the *ab initio* calculations, and we found that the hard and easy axis of magnetization were perpendicular to the C_2_ symmetry axis for Co(Pyipa)_2_, and Ni(Pyipa)_2_, respectively.

Before grafting the molecules onto iron oxide, we checked their integrity by performing X-ray photoelectron spectroscopy (XPS) on crystalline samples dissolved in methanol and casted on a graphite substrate (HOPG). We measured the XPS of a fresh and of a 2-day incubated solution (as in the case of the grafting procedure on the magnetic substrate). The XPS spectra at the N1s and P2p edges are almost identical for the four samples (Supplementary Figs 3–6; Supplementary Table 2). The analysis of the spectra allowed quantification of the relative percentages of three elements (M, N and P) for each sample, which is in agreement for the expected theoretical ratio 1:4:2 for M:N:P (Supplementary Table 2; Supplementary Discussion: ‘XPS analysis’).

### Monolayer preparation and characterization

**Figure 3:**
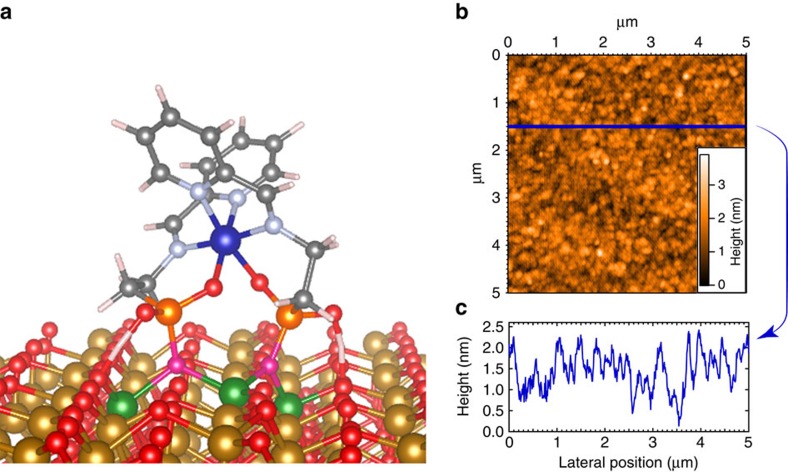
Monolayer of Co(Pyipa)_2_ on epitaxial Fe_3_O_4_. (**a)** Schematic representation of the DFT minimized orientation of Co(Pyipa)_2_ anchored onto epitaxial Fe_3_O_4_: C, grey; N, lilac; O, red or magenta when bound to a phosphonate; P, orange; H, white; Co, blue; Ni, green; Fe, gold or green when bound to the molecule; (**b**) AFM image of a monolayer of Co(Pyipa)_2_ anchored onto epitaxial Fe_3_O_4_; (**c**) Surface profile of the monolayer of Co(Pyipa)_2_.

A monolayer of molecules (Co- or Ni(Pyipa)_2_) on epitaxial Fe_3_O_4_ (111) was obtained by self-assembly from solution (see Methods for details). This reaction is well documented and has been widely used^17^,^20^,^21^,^22^,^23^,^24^, and proceeds in the following manner: the hydroxyl groups present on the surface are protonated by the phosphonic acid of the complex leading to PO^−^ species that react with the iron ions of the substrate causing the expulsion of water molecules (Fig. 1)^17^. We monitored surface coverage by atomic force microscopy (AFM) and time-of-flight secondary ion mass spectrometry (ToF-SIMS) (Fig. 3; Supplementary Figs 7–10). AFM data revealed homogenous coverage of the substrates (Fig. 3b; Supplementary Fig. 7) with the surface profile indicating a height of the objects that is consistent with the complexes being deposited onto the surface (∼1 nm). By comparing the AFM images before and after surface deposition (Fig. 3b; Supplementary Fig. 7a), we note the absence of degradation of the surface (we do not observe holes or aggregates), and we note an increase in the height of the surface profile, which is consistent with a monolayer on the surface. We, however, wanted to use a different and complementary technique to ensure the formation of chemical bonds between the complexes and the substrate. We thus performed Tof-SIMS studies that allowed detecting the molecular fragments confirming the presence of the molecules on the iron oxide substrates. Positive and negative ToF-SIMS spectra (Supplementary Figs 8–10; Supplementary Tables 2–3) showed a variety of fragments confirming that the phosphonic acid moiety binds to the iron oxide via the oxygen (Fe-O-P(OR)_2_) as assumed. It also showed larger fragments (Co(Pyipa)-Fe, Ni(Pyipa)-Fe) indicating that the ligand is bound to both the iron of the surface and the metal ion of the complex. Particularly, the detection of fragments such as [M(C_5_H_4_NCHN)_2_]^+^, [M(PO_3_)_2_FeO_2_]^−^, and related ones (Supplementary Figs 8–10; Supplementary Tables 3–4) that contain parts of the two tridentate ligands linked to M confirms that the intact complexes (M and the two tridentate ligands) are chemically linked to the substrate. It is important to note that if the phosphonates were weakly bound to the iron oxide surface (that is, hydrogen bonded or mere physical adsorption), we would not expect complex fragments containing both iron and phosphonate to be present. Similar fragmentation was found in ToF-SIMS data of octadecylphosphoric acid on tantalum oxide surfaces^22^. The above-mentioned observations indicate that our molecules remain intact during the deposition process, and that they chemically bind to the surface *via* the phosphonate group. Of course, we could have some unreacted molecules present, but we believe that our thorough washing steps (coupled with absence of aggregates in the AFM images) are sufficient to remove all unreacted molecules from the surface. The XPS spectra of monolayers of complexes were too noisy to be quantitatively analysed. However, X-ray absorption (XAS) spectra of bulk (on graphite) and grafted monolayers are identical (see below); the presence of large amount of damaged molecules linked to the substrate will lead to different XAS spectra for the bulk and grafted samples. In addition, the observation of the angular dependence of the magnetic behaviour of the Co-grafted substrate (see below) excludes the presence of damaged molecular fragments that would be statistically oriented on the surface. In summary, the combination of the results from the three techniques (XPS, TOF-SIMS and XAS) ensures that the Co- and Ni-containing complexes keep their integrity when reacted with the Fe_3_O_4_ substrates and that are indeed chemically linked to the surface.

### DFT calculations of the surface

Since the epitaxial Fe_3_O_4_ (111) surface has three different sites with O-O distances close to that of the OH–OH separation of the complexes (see structure description of the molecules above), the preferential coordination sites of the substrate together with the orientation of the molecules was determined by numerical simulations^17^. Determination of the equilibrium state and energy minimum of the system has been performed using density functional theory (DFT) molecular dynamics technique (FIREBALL)^28^,^29^,^30^,^31^. In our calculations, we set the molecule onto a surface of Fe_3_O_4_ oriented along the (111) direction formed by octahedral (O_h_) and tetrahedral (T_d_) Fe ions bridged by oxo ligands^32^. We initially positioned the molecule in three different configurations (a physisorbed and two chemisorbed ones, see SI for details). The orientation of the molecule was chosen such that the anchoring phosphonate moieties pointed towards the surface with the different possible binding modes^17^. Since the physisorbed energy is not compatible with the experimental rinse cycles (the molecular layer is stable after this operation), we decided to directly simulate the anchoring of the molecule by substituting the surface oxygen atoms by the phosphonates one (this configuration is otherwise out of range of standard DFT optimization due to a strong potential barrier between the physisorbed and an anchored state). We proceeded to do a DFT molecular dynamic simulation at room temperature to reproduce the experimental conditions of molecular deposition on the surface, followed by a structural optimization at 0 K to determine the final configuration on the surface. This procedure gave a stable structure and the molecular adsorption energies of the three configurations (Supplementary Discussion: ‘Density Functional Theory–Geometrical optimization of the complexes on the surface’)^33^. Further magnetic optimization was performed on these structures to obtain an accurate description of the magnetic state of each molecule. The most stable orientation corresponds to the condition where the C_2_ symmetry axis of the complexes is perpendicular to the surface as expected from the orientation of the oxygen phosphonate anchoring groups (Fig. 3a); the two oxygen atoms belonging to the two different phosphonates (phos) have replaced two oxygen atoms (bridging octahedral and tetrahedral Fe ions) belonging to the surface creating Fe–O_phos_ coordination bonds. This configuration leads to the molecules being linked to three Fe atoms through O_phos_ (Fe_oct_ –O_phos_–Fe_tet_–O_phos_–Fe_oct_) , as highlighted in Figs 1 and 3a. It is important to note that there are three possible anchoring sites for the complexes on the Fe_3_O_4_ (111) surface with O–O distances of 2.85, 2.97 and 3.08 Å. The most stable situation was found for the molecules bound on the site with O–O distance of 2.85 Å, which is reasonable because the distance between the two oxygen atoms of the phosphonic acid groups (O_phos_) is 2.59 Å. In addition, the length of the molecule measured perpendicular to the C_2_ axis (7.5 Å) excludes its grafting on all the binding sites and ensures an almost total coverage of the surface even if only one over six sites in average are occupied with a complex. For Ni(Pyipa)_2_, the easy *axis* of magnetization found for the isolated complex thus is parallel to the surface, while the hard axis found for the isolated Co(Pyipa)_2_ complex is parallel to the surface and thus the easy plane is perpendicular to the surface.

### Magnetic characterization of the monolayers

**Figure 4:**
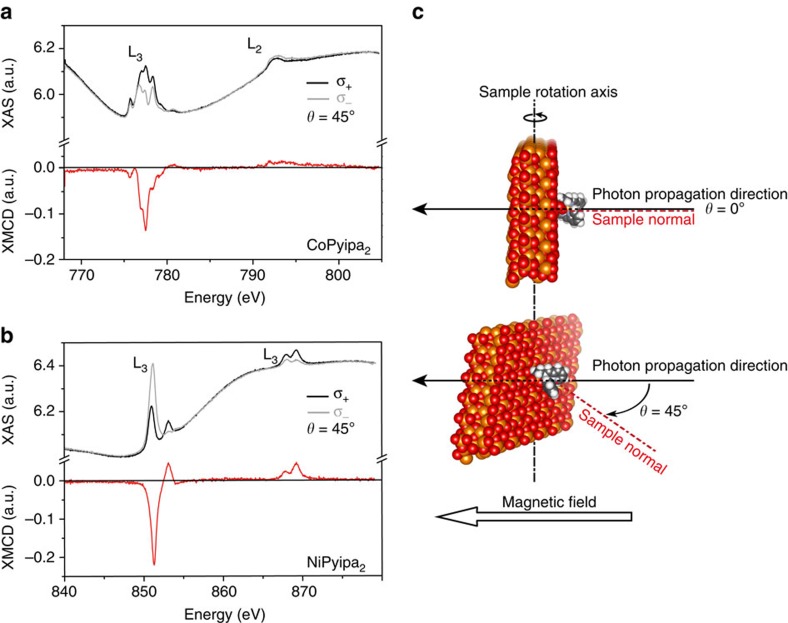
XAS/XMCD spectra of a monolayer of Co- and Ni(Pyipa)_2_. (**a**) Cobalt, and (**b**) Nickel L_2,3_ edges XAS (black and grey line) and XMCD (red lines) spectra recorded at *T*=2 K, and *θ*=45° using left (σ_+_) and right hand (σ_−_) circularly polarized light in 6.5 T field; (**c**) schematic representation of the measurement geometry.

To attain the sensitivity required to probe a monolayer of molecules, element specific magnetic properties were investigated by recording the magnetic dichroic component of the XAS of the surface (Fe) and of the complexes (Co and Ni for Co- and Ni(Pyipa)_2_, respectively) at the L_2,3_ edges. Circular polarized X-rays were employed for two incidence angles (*θ*=0° and 45°) between the sample normal and the X-ray propagation vector. All measurements were recorded at 2 K in the total yield electron mode with the magnetic field aligned parallel to the photon propagation vector^7^,^34^. The XAS spectra, and the resulting XMCD spectra (σ^−^-σ^+^), at the iron, cobalt and nickel L_2,3_ edges were acquired in the presence of a 6.5 T field employing the two circular polarizations (σ^+^; σ^−^). The XAS and XMCD spectra at the Fe L_2,3_ edges of the epitaxial Fe_3_O_4_ surface before and after molecule grafting are similar (Supplementary Figs 11 and 12), and are comparable to those published in the literature^35^, demonstrating that the surface deposition method did not damage the substrates. The XAS and XMCD spectra at the Ni and the Co edges show a strong dichroic signal that is indicative of molecules deposited on the surface (Fig. 4; Supplementary Fig. 13). Note that the non-flat background for the XMCD for Co comes from the XMCD signal of the Fe L_2,3_ edges and can be removed by recording the XMCD on a pure Fe_3_O_4_ surface in the energy range of the Co L_2,3_ edges as shown in Supplementary Fig. 14. The signals were compared with those recorded on a thick film of the same complexes deposited by drop casting (Supplementary Fig. 15). The spectra are similar, and confirmed that the electronic structure of the molecule is retained upon grafting. The shape of the XMCD signal at the Fe L_3_ edge (Supplementary Fig. 12) corresponds to the situation where the magnetic moments of the octahedral Fe sites are aligned with the magnetic field (negative XMCD signal) and are antiparallel to those of the tetrahedral sites (positive XMCD signal), which correspond to an antiferromagnetic coupling between octahedral and tetrahedral sites^35^. The XMCD signal at the Co L_3_ edge is aligned with the magnetic field (negative XMCD), therefore, it is aligned with the octahedral Fe sites. The identical scenario is true for Ni.

A sum rule analysis^36^,^37^ was performed to extrapolate the values of the spin and orbital magnetic moments for Co and Ni in the complex deposited as a monolayer on the Fe_3_O_4_ surface at *θ*=0° and 45°, and as a thick film at *θ*=45°. This analysis allows to obtain the spin and orbital magnetic moments from the integrated XMCD signals, and gave the results summarized in Table 2 (see Supplementary Information for the details of the calculations). Interestingly the Co magnetic moment found corresponds to one third of the saturation magnetization of 3 *μ*_B_ for an isotropic *S*=3/2 cobalt ion. This result confirms that the ground state of the Co^II^ ion is mainly built from *M*_*S*_=± 1/2 with very small contributions from higher *M*_*S*_ (± 3/2) terms, exactly what was found from the magnetization measurements analysis (Supplementary Discussion: ‘Sum rule calculations’). It is important to note that X-ray sum rule analysis assumes a transition between two well-defined shells (that is, 2*p* to 3*d* valence state transition for transition metal ions), and that the 3*d* valence states are separable from the other state. Also, from the literature^38^ we do not expect a large deviation of the values of <*T*_z_> (magnetic dipole term) as the complexes are hexacoordinated so no real structural anisotropy is present. Thus, we neglected this term in our calculations.

**Figure 5:**
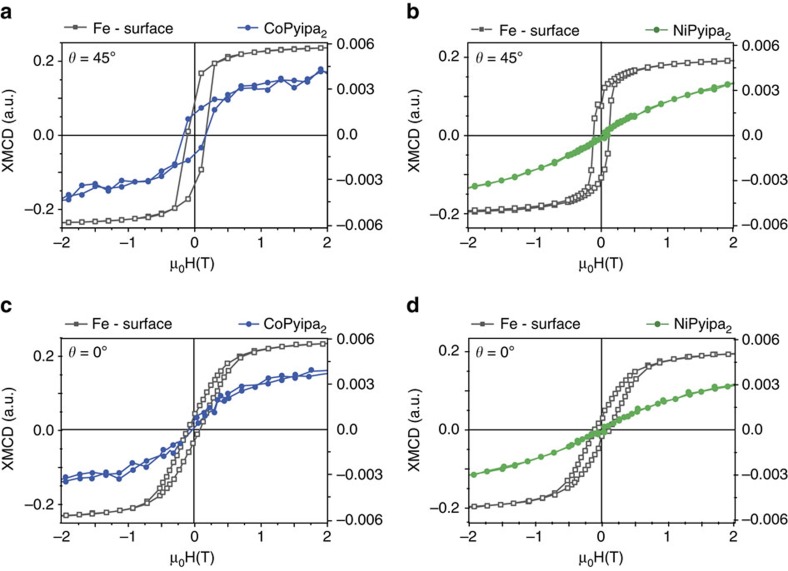
Element-specific field dependence of the magnetization of the complexes and surface (Fe). Hysteresis curves (multiplied by −1) of the Co atoms (blue), Ni atoms (green) and Fe atoms (grey) obtained at the L_2,3_ edges XMCD maxima at *T*=2 K, and *θ*=45°. (Monochromatized X-rays are set at the energy of the maximum absolute value of the XMCD signal (that is, *hν*=777.5 eV for Co, *hν*=851 eV for Ni, and *hν*=707 eV for Fe) then the external magnetic field is switched step by step from +6.5 T down to −6.5 T and back to +6.5 T. At each step the magnetic field is switched from left to right circular polarization to yield the element specific magnetization curves.

To gain insight on the nature of the interaction of Co- and Ni(Pyipa)_2_ with the epitaxial Fe_3_O_4_ surface, we measured element-specific XMCD-detected hysteresis loops. The field-dependence Fe, Co and Ni L_3_ XMCD intensity (multiplied by −1) at fixed photon energy and normalized are shown in Fig. 5 and Supplementary Fig. 16 for the geometry where the X-ray propagation vector is at 45° and 0° with the sample normal i.e. the substrate makes an angle of 45° and 0° with the applied magnetic field (Fig. 3c). It was possible to observe an opening of the magnetic hysteresis loop for Co(Pyipa)_2_ (Fig. 5a), while no opening occurs when the magnetic field is perpendicular to the substrate (Fig. 5c; Supplementary Fig. 16a). These results indicate that the Co-containing complex is magnetically coupled to the Fe_3_O_4_ (111) surface and that the molecules are magnetically oriented otherwise no angular dependence of the magnetization would be observed. To investigate the magnetic behaviour of Co(Pyipa)_2_ isolated from the Fe_3_O_4_ (111) surface, an amorphous non-magnetic ultrathin insulating layer (1 nm of Al_2_O_3_) was deposited between the iron oxide surface and the molecules. It is important to note that the same type of reactivity is possible on Al_2_O_3_ layer as for Fe_3_O_4_ and other oxides but no structural orientation of the molecules is expected because the Al_2_O_3_ layer is amorphous^17^,^20^,^21^,^22^,^23^,^24^. In this system, the XAS and XMCD spectra are similar to those when the molecules are grafted on iron oxide (Supplementary Fig. 17), but no opening of the magnetization loop was seen for *θ*=45° (Supplementary Fig. 18). These data indicate that the hysteresis loop for the Co/Fe_3_O_4_ system is due to the magnetic coupling between the molecules and the iron-oxide because in the presence of the diamagnetic Al_2_O_3_ where no magnetic coupling is expected no hysteresis loop is observed.

Furthermore, since the loop is open at 45°, the easy plane of magnetization of the anchored complex is parallel to the substrate or the anisotropy nature of the molecule has changed upon coupling to have an easy axis of magnetization that is parallel to substrate. The experimental data cannot rule out between these two possibilities: if the anchored complexes had their easy axes of magnetization parallel to the surface, the magnetic behaviour would be the same as if each molecule had an easy plane parallel to the substrate because the azimuthal distribution of the easy axes. This last hypothesis implies that the nature of the magnetic anisotropy of the complex has changed upon coupling with the substrate from an easy plane to an easy axis (parallel to the substrate). While the first hypothesis implies that the complex kept its anisotropy nature (easy plane of magnetization that contains its C_2_ symmetry axis when isolated, Fig. 3a) but switched its orientation to be perpendicular to the C_2_ axis (thus parallel to the substrate) upon coupling with the magnetic substrate. This interpretation is supported by the values of the magnetic moment extracted from the sum rule applied on Co for the two orientations (Table 2) where a slight increase is observed for the 45°. A precise determination of the anisotropy tensor axes and to rule out one of the two hypotheses require further studies at several orientation of the magnetic field. No opening of the magnetization loop was seen for Ni(Pyipa)_2_ (Fig. 5b,d; Supplementary Fig. 16b) for the two orientations highlighting the absence of (or the presence of very weak) magnetic coupling with the substrate. We reasoned that the difference in coupling between Ni(Pyipa)_2_ (absence of hysteresis) and Co(Pyipa)_2_ (presence of hysteresis) excludes dipole–dipole interactions between Co(Pyipa)_2_ and substrate, as such interactions would have resulted in the same behaviour for the two molecules. The magnetic coupling between Co(Pyipa)_2_ and Fe_3_O_4_ thus is due to exchange.

### Broken symmetry calculations

**Figure 6:**
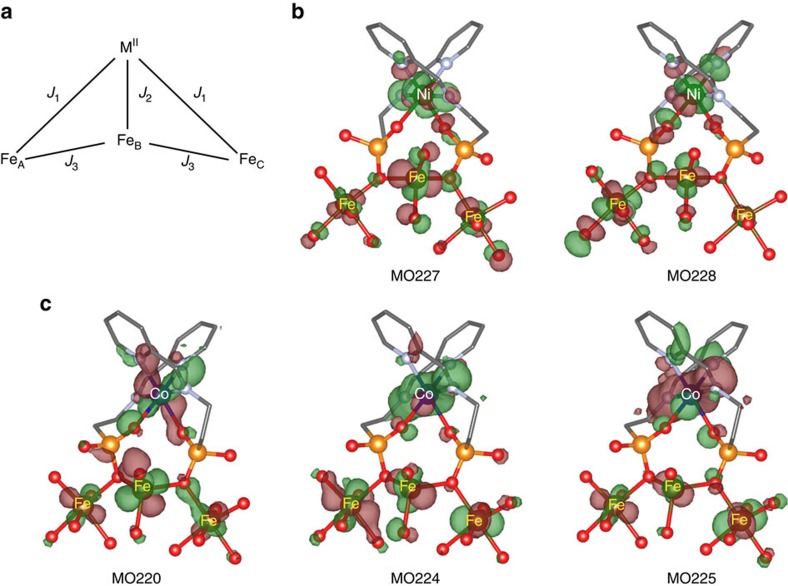
Geometry of the exchange interaction. (**a**) Interaction topology between the MPyipa_2_ (M=Ni or Co) and the surface Fe ions. Optimized geometry of the singly occupied magnetic orbitals for Ni(Pyipa)_2_ (**b**) and Co(Pyipa)_2_ (**c**). Positive (red) and negative (green) isosurfaces for the high-spin solution. C, grey; N, lilac; O, red; P, orange; Co, blue; Ni, green; Fe, dark orange; hydrogen atoms were omitted for clarity. The MO labels are in ascending energy levels.

To elucidate the nature and relative magnitude of this exchange coupling, and the differences between the magnetic behaviour of Co- and Ni(Pyipa)_2_ when anchored to the surface, we carried out broken symmetry calculations using the B3LYP functional of the GAUSSIAN 09 package (Supplementary Discussion: ‘Density Functional Theory – calculation of the magnetic coupling between the molecules the surface’)^39^,^40^,^41^,^42^,^43^. We employed the extended basis sets: Valence Triple Zeta plus Polarization (VTZP) for Fe, Ni, Co and for the ligand atoms^44^,^45^,^46^. Starting from the results of the DFT molecular dynamic simulation described above, we optimized the geometries of the complexes and their anchoring to the surface. The system is composed of the three coupled Fe^III^ ions (*S*=5/2) (Fe^III^_Oh_—Fe^III^_Td_–Fe^III^_Oh_) as explained above, we assume that the surface Fe^II^ atoms were oxidized to Fe^III^)^47^ surrounded by their coordinated oxygen atoms (from the surface) and the complexes either Co(Pyipa)_2_ (Co^II^, *S=*3/2) or Ni(Pyipa)_2_ (Ni^II^, *S=*1). The Heisenberg Hamiltonian 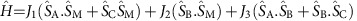 describes the low energy spectrum of the four spin centres: M=Ni^II^ or Co^II^, 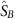
=the spin operator of the central tetracoordinated 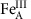 ion, 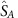 and 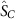
=the spin operators of its hexacoordinated neighbors 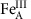 and 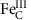, respectively (Fig. 6a). The analytical energies of the four possible computed solutions are given in Supplementary Fig. 19. The Hamiltonian was chosen so that when the exchange parameters (*J*_i_) are positive the coupling is antiferromagnetic.

First, we calculated the surface coupling in the absence of Co- or Ni(Pyipa)_2_ (*J*_1_=*J*_2_=0). As expected for an epitaxial Fe_3_O_4_ (111) surface, we found that the surface Fe^III^ have an antiferromagnetic coupling (*J*_3_=52 cm^−1^). The ferromagnetic solution was 1,321 cm^−1^ higher in energy than the antiferromagnetic one. Second, we computed the energies in the presence of Co- or Ni(Pyipa)_2_.

By computing the energies of the different solutions based on the coupling scheme depicted in Fig. 6a, we find that the coupling between the Ni^II^(or Co^II^) ions and the surface is independent from the nature (ferromagnetic or antiferromagnetic) of the exchange coupling within the substrate (Supplementary Discussion: ‘Density Functional Theory–calculation of the magnetic coupling between the molecules the surface’). From the energy differences between the various solutions (Supplementary Table 5) we obtained *J*_1_=18 cm^−1^, *J*_2_=–2 cm^−1^, *J*_3_=–40 cm^−1^ for Ni(Pyipa)_2_, and *J*_1_=–54 cm^−1^, *J*_2_=–49 cm^−1^; *J*_3_=–47 cm^−1^ for Co(Pyipa)_2_. This translates into a weakly antiferromagnetic exchange coupling between the Ni(Pyipa)_2_ and the surface and a strongly ferromagnetic coupling for Co(Pyipa)_2_. It is worth noting two important points. (1) The values of *J* are not exact values, but rather their magnitude should be considered. (2) The calculations suggest that the exchange between tetrahedral and octahedral Fe ions became ferromagnetic upon grafting. It is unfortunately difficult to confirm this theoretical result from the present experimental data because, due to the size of the molecule, only a weak percentage of the Fe ions are linked to the complexes; dedicated specific measurements comparing bare and grafted samples are needed to reach an unambiguous conclusion.

The opposite nature of the exchange interaction can be explained by the difference in the molecular orbitals (MO) depicted in Fig. 6. None of the singly occupied molecular orbitals (SOMOs) of Co(Pyipa)_2_ have a strong overlap with the Fe^III^ SOMOs through the bridging oxygen ligands (see MOs number 220, 224 and 225 in Fig. 6). The most important physical factor contributing to the exchange coupling of Co(Pyipa)_2_ with the surface is brought by the direct exchange integrals between all the magnetic SOMOs, which are always ferromagnetic^48^,^49^, and the spin polarization. On the contrary, one of the two SOMOs of Ni(Pyipa)_2_ (MO number 228 (Fig. 6)) exhibits an extended delocalization tail on the oxygen atom bridging the complex to the surface through the phosphonate anchoring group. The overlap between this Ni(Pyipa)_2_ SOMO and the Fe^III^ one brings a large kinetic contribution (antiferromagnetic) to the exchange coupling that compensates and overcomes the ferromagnetic contribution and leads to an overall antiferromagnetic interaction, though weaker in magnitude than the ferromagnetic one in the case of Co (refs 48, 49). As the magnetic orbitals of the Ni(II) ion are lower in energy than those of the Co(II) ion, the interaction between the occupied orbitals of the ligand and those of the metal ions are stronger for the Ni(II) complex than for the Co(II) one. For this reason, the Ni(II) ion magnetic orbitals (of σ nature) are more delocalized over the ligands than those of the Co(II) ion. As these interactions mediate the coupling between the complexes and the surface, a stronger antiferromagnetic contribution to the magnetic coupling is expected for the Ni(II) complex, thus compensating the ferromagnetic contribution and leading ultimately to an overall weaker magnetic coupling than for Co(II). The difference between the nature and the magnitude of the couplings is also corroborated by the shorter M^II^/spacer/surface distance for Ni(Pyipa)_2_ than for Co(Pyipa)_2_, strengthening the antiferromagnetic superexchange mechanism. It is important to note that the absolute numerical values of *J*_i_ have to be considered with caution due to the approximations made in the calculations. The much stronger ferromagnetic coupling of the Co^II^ centre with the surface compared with the Ni^II^ centre explains the opening (or lack thereof) of the magnetic hysteresis for Co(Pyipa)_2_ (or Ni(Pyipa)_2_) when deposited onto the ferrimagnetic surface. XMCD measurements at lower temperatures (XMCD below 1 K is not accessible on the DEIMOS line) would likely provide further indications on the nature of the magnetic coupling in the Ni(Pyipa)_2_ system^7^,^8^.

## Discussion

The experimentally observed change in the anisotropy tensor of the Co-containing molecules is mainly due to exchange coupling with the ferromagnetic substrate and not to a change of the local structure of the molecules that might have occurred upon grafting. The following three points support this conclusion: (i) the local structure of the grafted molecules, optimized by DFT, shows no important changes in the metal-ligand bond distances and angles. (ii) The zero-field splitting value of the isolated molecules is relatively large (+30 cm^−1^), and only a large change of the local Co environment is necessary to alter the single ion anisotropy tensor. (iii) The magnitude of the exchange coupling between the Co-containing complex and the substrate (computed by DFT) is of the same order of magnitude as the anisotropy energy of the isolated complex; an alteration of the anisotropy tensor of the isolate molecules is thus expected due to exchange coupling as experimentally observed. If the exchange coupling were much weaker than the anisotropy energy of the isolated molecules, no change in the single ion anisotropy tensor of the isolated molecules would have been detected.

In conclusion, we have shown that by molecular design, we can chemically anchor metal-containing molecules to a magnetic iron oxide electrode and we can finely control the molecule/magnetic surface interface structure. The coupling between the magnetic molecules and Fe_3_O_4_ is due to exchange and not to dipole–dipole interactions. The chemical anchoring responsible of the exchange magnetic coupling switches the orientation of the easy plane of magnetization of the Co(II) complex or changes the nature of its magnetic anisotropy from an easy plane to an easy axis of magnetization. The electronic structure of the metal ion has a paramount importance on the nature of the coupling: Co(Pyipa)_2_ undergoes a strong ferromagnetic coupling with the substrate while the structurally analogous Ni(Pyipa)_2_ is either not coupled or weakly antiferromagnetically coupled to the substrate. We can, therefore, envision that controlling the molecule/substrate exchange interaction would allow tuning the spin current (sign of the magneto-resistance) at the interface and eventually lead to using such current to switch the magnetization of single anisotropic molecules in molecule-based spintronic devices.

## Methods

### General

Epitaxial Fe_3_O_4_(111) thin films were grown by molecular beam epitaxy on α-Al_2_O_3_ (0001) according to literature procedures^50^. Unless otherwise stated, all reagents were purchased from Aldrich or TCI and used without further purification. Electrospray ionization mass spectrometry (ESI-MS) spectra were recorded on a Thermo Scientific 2009 mass spectrometer. IR spectra were recorded on a Bruker TENSOR-27 Fourier transform infrared (FT-IR) spectrometer equipped with an attenuated total reflectance (ATR crystal diamond/ZnSe) sample holder in the 4000–500 cm^−1^ range. Elemental analysis was taken on a Thermo Scientific Flash analyzer.

### Synthesis of Co(Pyipa)_2_

(Cobaltl(II) bis-2-[(Pyridin-2-ylmetylene)amino]ethyl phosphonic acid). A round bottom flask was charged with 2-aminoethylphosphonic acid (77.8 mg, 0.622 mmol), 2-pyridinecarboxaldehyde (58 μl, 0.610 mmol), Co(CH_3_COO)_2_·4 H_2_O (75 mg, 0.301 mmol), MeOH (4 ml) and demineralized H_2_O (4 ml). The reaction mixture was heated at reflux for 3 h. The solvent was evaporated and a red product was obtained. The product was purified by vapor diffusion of Et_2_O into a MeOH solution of Co(Pyipa)_2_ (*m=*109.5 mg; yield=74%). IR (ν/cm^−1^): 3,317 (br), 2,946 (br), 2,888 (br), 2,529 (br), 2,162 (s), 1,981 (br), 1,780 (m), 1,660 (s), 1,626 (m), 1,589 (m), 1,571 (m), 1,469 (br), 1,446 (s), 1,366 (br), 1,352 (br), 1,286 (s), 1,226 (s), 1,156 (s), 1,104 (m), 1,091 (m), 1,051 (m), 1,033 (m), 1,010 (s), 988 (s), 962 (m), 937 (s), 897 (s), 875 (s), 813 (m), 785 (m), 744 (m), 707 (br), 662 (s), 634 (m). ESI-MS: *m/z* 486.03 ([Co(Pyipa)_2_]^+^). Elem anal. Calcd for C_16_H_24_N_4_O_8_P_2_Co: C, 36.87; H, 4.64; N, 10.75. Found: C, 36.80; H, 4.82; N, 10.73.

### Synthesis of Ni(Pyipa)_2_

(Nickel(II) bis-2-[(Pyridin-2-ylmetylene)amino]ethyl phosphonic acid). A round bottom flask was charged with 2-aminoethylphosphonic acid (85.0 mg, 0.680 mmol), 2-pyridinecarboxaldehyde (64 μl, 0.673 mmol), Ni(CH_3_COO)_2_·4 H_2_O (80 mg, 0.321 mmol), MeOH (4 ml) and demineralized H_2_O (4 ml). The reaction mixture was heated at reflux for 3 h. The solvent was evaporated and a red product was obtained. The product was purified by vapor diffusion of Et_2_O into a MeOH solution of Ni(Pyipa)_2_ (*m=*98.6 mg; yield=67%). IR (ν/cm^−1^): 3,317 (br), 2,946 (br), 2,888 (br), 2,529 (br), 2,162 (s), 1,981 (br), 1,780 (m), 1,660 (s), 1,626 (m), 1,589 (m), 1,571 (m), 1,469 (br), 1,446 (s), 1,366 (br), 1,352 (br), 1,286 (s), 1,226 (s), 1,156 (s), 1,104 (m), 1,091 (m), 1,051 (m), 1,033 (m), 1,010 (s), 988 (s), 962 (m), 937 (s), 897 (s), 875 (s), 813 (m), 785 (m), 744 (m), 707 (br), 662 (s), 634 (m). ESI-MS: *m/z* 485.04 ([Ni(Pyipa)_2_]^+^). Elem anal. Calcd for C_16_H_28_N_4_O_10_P_2_Ni: C, 34.50; H, 5.07; N, 10.06. Found: C, 34.37; H, 4.54; N, 10.02.

### Single crystal X-ray diffraction studies

X-ray diffraction data were collected by using a Kappa X8 APPEX II Bruker diffractometer with graphite-monochromated Mo_Kα_ radiation (*λ*=0.71073 Å). Crystals were mounted on a CryoLoop (Hampton Research) with Paratone-N (Hampton Research) as cryoprotectant and then flashfrozen in a nitrogen-gas stream at 100 K. The temperature of the crystal was maintained at the selected value (100 K) by means of a 700 series Cryostream cooling device to within an accuracy of ±1 K. The data were corrected for Lorentz polarization, and absorption effects. The structures were solved by direct methods using SHELXS-97 (ref. 51) and refined against *F*^2^ by full-matrix least-squares techniques using SHELXL-97 (ref. 52) with anisotropic displacement parameters for all non-hydrogen atoms. Hydrogen atoms were located on a difference Fourier map and introduced into the calculations as a riding model with isotropic thermal parameters. All calculations were performed by using the Crystal Structure crystallographic software package WINGX^53^.

### Molecular magnetic measurements

The magnetic susceptibility measurements were obtained using a Quantum Design SQUID magnetometer MPMS-XL7 operating between 1.8 and 300 K for d.c.-applied fields ranging from −5 to 5 T. Dc analysis was performed on polycrystalline samples of Co- and Ni(Pyipa)_2_ (17.61 mg and 17.31 mg, respectively) wrapped in eicosan under a field between 0.1 and 1 T and between 1.8 and 300 K. The *χ*_*M*_*T=f*(T) curve for Ni(Pyipa)_2_ shows a Curie-law behaviour between 300 and 50 K (ground state with no first order orbital momentum ^3^A_2g_) and then decreases indicating the presence of a zero-field splitting (ZFS) within the *S*=1 state (Supplementary Fig. 1). While, for Co(Pyipa)_2_ a steady decrease is observed from room temperature down to 75 K and then more rapidly, in line with a ground state with a non completely quenched orbital momentum as expected for slightly distorted octahedral for Co^II^ complexes^54^. The data for Co- and Ni(Pyipa)_2_ were fitted by a full diagonalization of the energy matrices considering many orientations of the magnetic field. The best fits lead to the following parameters: *D*_Ni_=–5.2 cm^−1^, *E*_Ni_=0, *g*_Ni_=2.22, *R* (agreement factor)=2 × 10^-5^, *D*_Co_=+ 30.2 cm^−1^, *E*_Co_=5.0 cm^−1^, *g*_Co_=2.3, *R*=5 × 10^-4^. *D* and *E* are the axial and the rhombic ZFS parameters, and *g* is the Lande factor of the spin Hamiltonian =*gμ*_B_**H**·**S**+*D*[_*z*_^2^ – *S*(*S*+1)/3]+*E* (_*x*_^2^–_*y*_^2^). These parameters are related to the *D* tensor matrix elements by 2*E*=|*D*_*xx*_*–D*_*yy*_| and *D*=3*D*_*zz*_/3.

### Monolayer preparation

The monolayers were prepared by immersion of the Fe_3_O_4_ substrates into 10 ml of a freshly filtered 0.5 mM solution of either Co(Pyipa)_2_ or Ni(Pyipa)_2_ dissolved in MeOH. After 3 days of immersion the substrates were removed from the solution and rinsed thoroughly with neat solvent. All the monolayer preparation experiments were carried out at room temperature.

### Geometrical optimization of the geometry of the molecule on the surface

The FIREBALL package uses a localized optimized minimal basis set^29^ and theself-consistency is achieved over the occupation numbers through the Harris functional^30^. The LDA exchange-correlation energy is calculated using the efficient multi centre weighted exchange correlation density approximation (McWEDA).^31^

### X-ray photoelectron spectroscopy

was carried out using monochromatized Al K alpha1 X-rays (*hν*=1,486.6 eV), a hemispherical analyzer, and a channel plate detector. The spectrometer was calibrated at the Au 4f core level at a binding energy of 84 eV. Spectra were recorded at a takeoff angle of 90°. The pass energy was set to 160 eV for survey and 20 eV for core level, giving an energy resolution of 0.38 eV.

### Atomic force microscopy

AFM images were recorded using a Pico-LE microscope (Molecular Imaging-Agilent Technologies) in contact mode. AFM tips were Si-coated with Pt/Ir alloy with a stiffness in between 0.1 N m^−1^ and 0.3 N m^−1^. The tip radius was given at 20 nm.

### Time-of-flight secondary ion mass spectrometry

Time-of-flight secondary ion mass spectrometry data were acquired using a TOF.SIMS V spectrometer (ION-TOF GmbH, Muenster, Germany). The analysis chamber was maintained at less than 5 × 10^−7^ Pa under operational conditions. The total primary ion flux was less than 10^12^ ions cm^-2^ ensuring static conditions. A pulsed 25 keV Bi^+^ primary ion source (Liquid Metal Ion Gun, LMIG) at a current of about 1 pA (high current bunched mode), rastered over a scan area of 500 × 500 μm was used as the analysis beam. Data acquisition and processing analyses were performed using the commercial IonSpec program. The exact mass values of at least seven known species, from H^−^, C^−^, C^−^_2_, C^−^_3_, PO_2_^−^, PO_3_^−^, FeO^−^, FeO_2_^−^, Fe_2_O^−^, FeOPO_3_^−^, and H^+^, CH_3_^+^, Na^+^, Ca^+^, Fe_2_^+^, C_5_H_5_Fe^+^, C_6_H_6_Fe^+^, were used for calibration of the data, acquired in the negative and positive ion mode, respectively.

### XAS/XMCD studies

The XAS/XMCD studies at the Fe, Co and Ni L_2,3_ edges were carried out at the DEIMOS beam line, SOLEIL Synchrotron (Gif-sur-Yvette, France)^55^,^56^. To ensure optimal detection sensitivity, the absorption spectra were measured in the Total Electron Yield mode. We used low density photons to avoid radiation damages to the samples. XMCD spectra were obtained from circularly polarized absorption spectra at 2 K under an applied magnetic field of 6.5 T parallel to the X-ray propagation vector. The XMCD-detected hysteresis loops were obtained at 2 K with the magnetic field sweeping (−6.5 to 6.5 T) parallel to the X-ray propagation vector.

### Data availability

The X-ray crystallographic coordinates for structures reported in this Article have been deposited at the Cambridge Crystallographic Data Centre (CCDC), under deposition numbers CCDC-1049643 and CCDC-1049644. These data can be obtained free of charge from The Cambridge Crystallographic Data Centre via www.ccdc.cam.ac.uk/data_request/cif. The remaining data that support the findings of this study are available from the corresponding authors upon reasonable request.

## Additional information

**How to cite this article:** Campbell, V.E. *et al*. Engineering the magnetic coupling and anisotropy at the molecule–magnetic surface interface in molecular spintronic devices. *Nat. Commun.* **7,** 13646 doi: 10.1038/ncomms13646 (2016).

**Publisher's note:** Springer Nature remains neutral with regard to jurisdictional claims in published maps and institutional affiliations.

## Supplementary Material

Supplementary Information Supplementary Figures 1-19, Supplementary Tables 1-5, Supplementary Discussion and Supplementary References.

Supplementary Data 1 CIF file for [Ni(Pyipa)_2_]

Supplementary Data 2 CIF file for [Co(Pyipa)_2_]

## Figures and Tables

**Table 1:** The experimental and calculated magnetization parameters reported in cm^-1^: *D*=axial zero field splitting parameter; *E*=rhombic zero field splitting parameter.

	**Co(Pyipa)**_**2**_	**Ni(Pyipa)**_**2**_
Experimental		
*D* (cm^−1^)	+30.2	−5.2
*E* (cm^−1^)	+5.0	0.0
Calculation		
*D* (cm^−1^)	+32.2	−3.4
*E* (cm^−1^)	+4.1	+0.7

**Table 2:** Values of the spin and orbital magnetic moments for Co and Ni in the complex deposited as a monolayer on the Fe_3_O_4_ surface at *θ*=0° and 45°, and as a thick film at *θ*=45°.

**Sample**	***M***_**L**_**=−*****μ***_**B**_**<L**_**Z**_**>**	***M***_***S***_**=−*****g***_**0**_***μ***_**B**_**<S**_**Z**_**>**	***M***_**L**_**/*****M***_***S***_
Co monolayer *θ*=0°	0.39±0.03 *μ*_B_	1.19±0.08 *μ*_B_	0.33±0.11 *μ*_B_
Co monolayer *θ*=45°	0.44±0.03 *μ*_B_	1.39±0.08 *μ*_B_	0.28±0.11 *μ*_B_
Ni monolayer *θ*=0°	0.35±0.02 *μ*_B_	1.39±0.12 *μ*_B_	0.25±0.14 *μ*_B_
Ni monolayer *θ*=45°	0.34±0.02 *μ*_B_	1.50±0.12 *μ*_B_	0.23±0.14 *μ*_B_
Co thick film *θ*=45°	0.44±0.03 *μ*_B_	1.25±0.08 *μ*_B_	0.36±0.11 *μ*_B_
Ni thick film *θ*=45°	0.17±0.02 *μ*_B_	1.34±0.12 *μ*_B_	0.13±0.14 *μ*_B_
