# Supplementary material for: Engineering the magnetic coupling and anisotropy at the molecule–magnetic surface interface in molecular spintronic devices
Source: Nat Commun. 2016 Dec 8;7:13646. doi: 10.1038/ncomms13646 (PMC5476799; doi:10.1038/ncomms13646)
Supplement: Supplementary Information — Supplementary Figures 1-19, Supplementary Tables 1-5, Supplementary Discussion and Supplementary References. [file ncomms13646-s1.pdf]

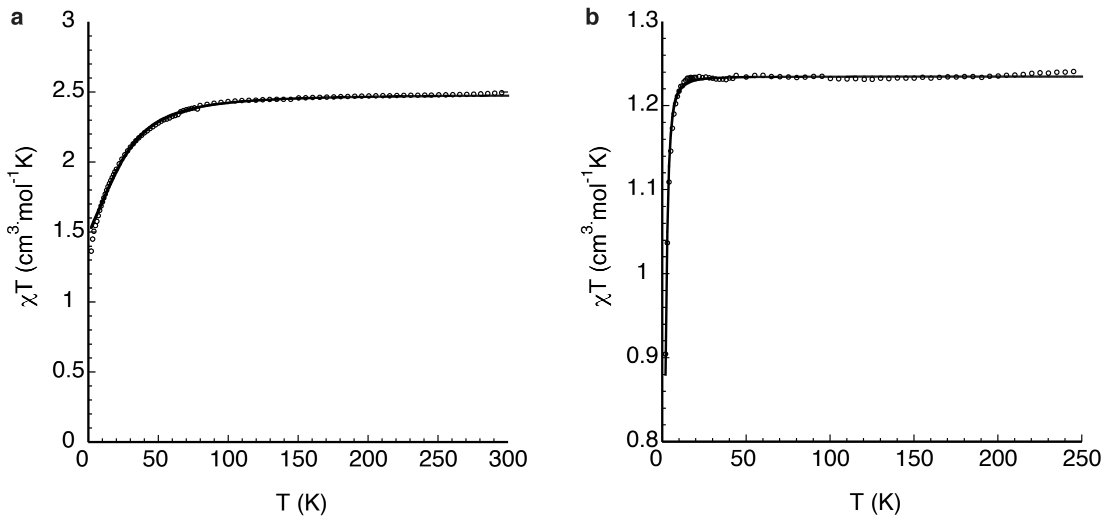

Supplementary Figure 1. $\chi_M T$ = f(T) curve for Co(Pyipa)$_2$ (a) and Ni(Pyipa)$_2$ (b). Solid line corresponds to the best fit.

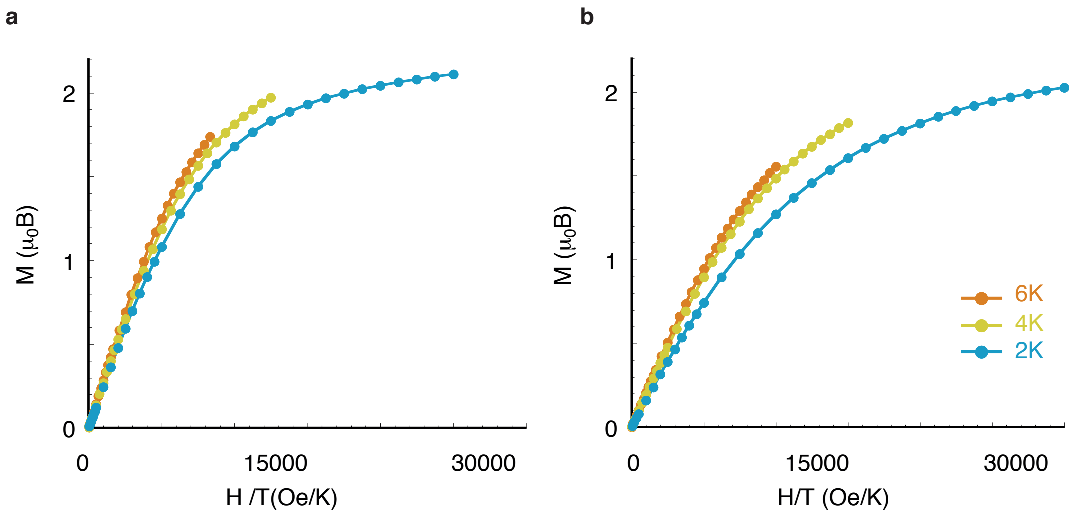

Supplementary Figure 2. Reduced magnetization plots ($M = f(\mu_0 H/T)$ for Co(Pyipa)$_2$ (a) and Ni(Pyipa)$_2$ (b).

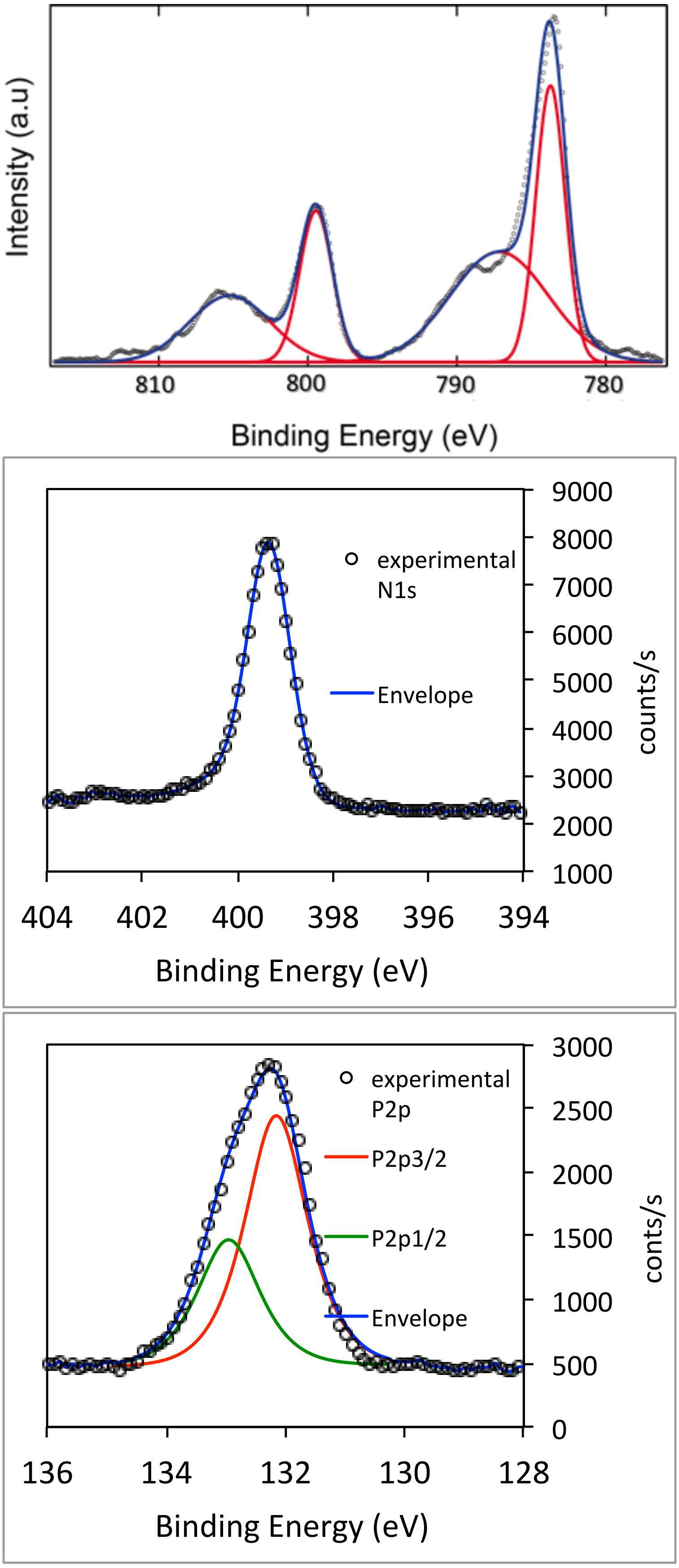

Supplementary Figure 3. XPS of Co((Pyipa))$_2$; at the Co2p edge (top), the N1s edge (middle) and the P2p edge (bottom).

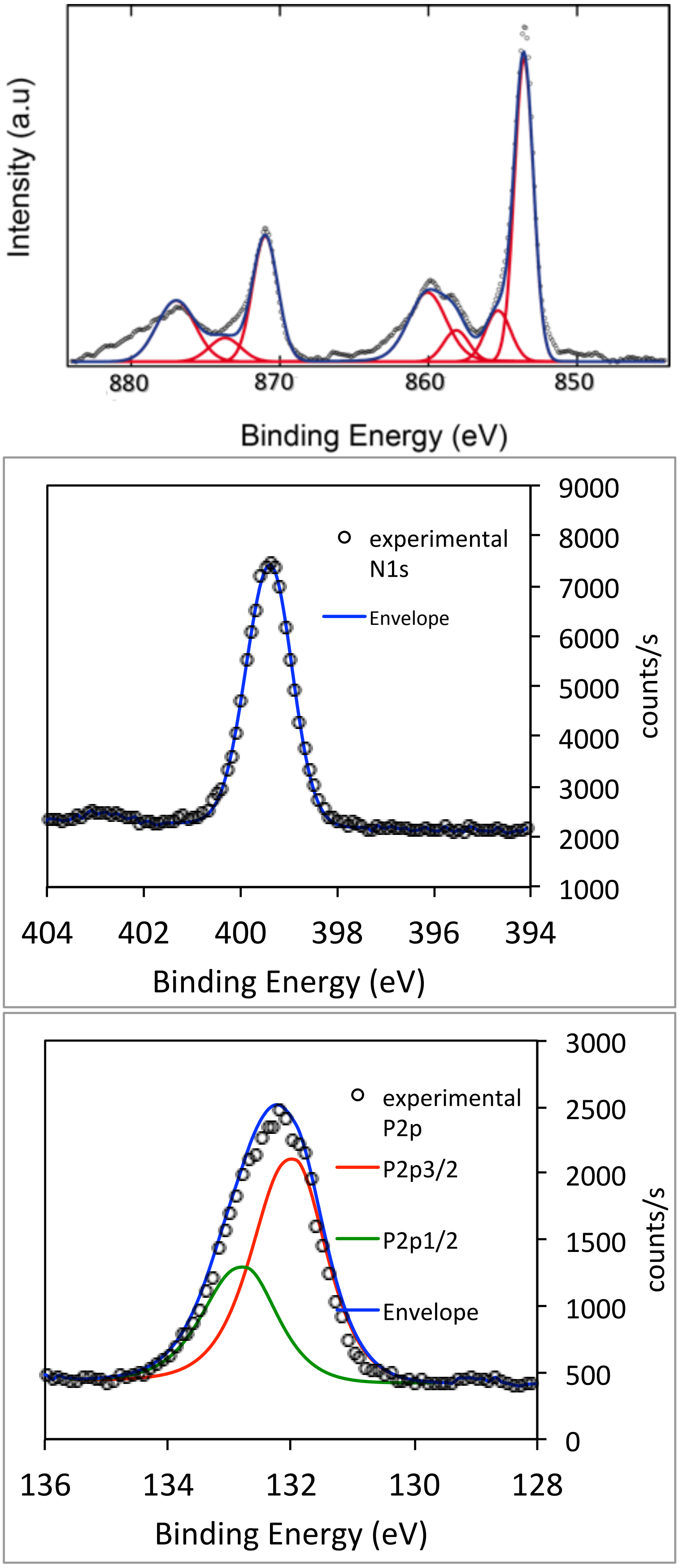

Supplementary Figure 4. XPS of Ni((Pyipa))$_2$; at the Ni2p edge (top), the N1s edge (middle) and the P2p edge (bottom)

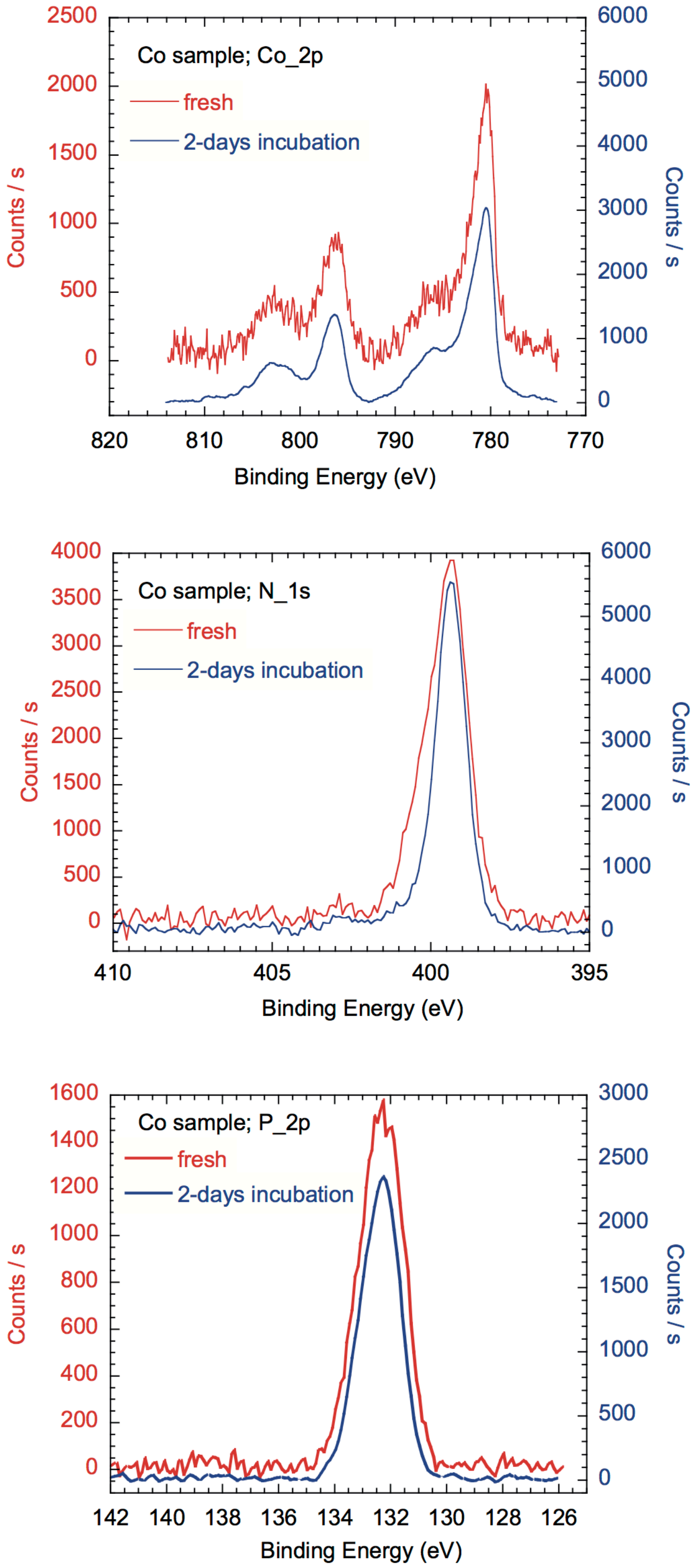

Supplementary Figure 5. XPS of Co((Pyipa))$_2$; at the Co2p edge (top), the N1s edge (middle) and the P2p edge (bottom) of fresh (red) and 2-day incubated (blue) samples.

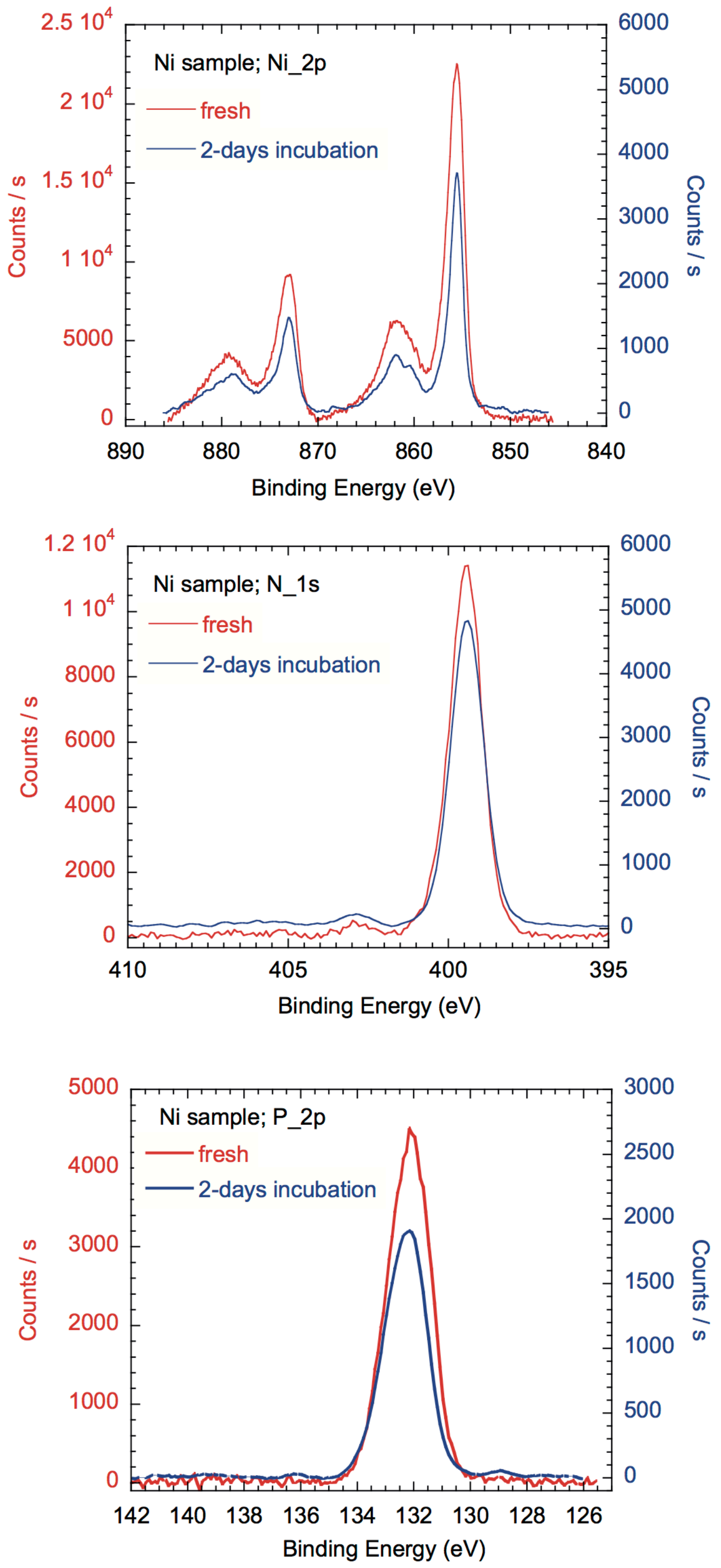

Supplementary Figure 6. XPS of Ni((Pyipa))$_2$; at the Ni2p edge (top), the N1s edge (middle) and the P2p edge (bottom) of fresh (red) and 2-day incubated (blue) samples.

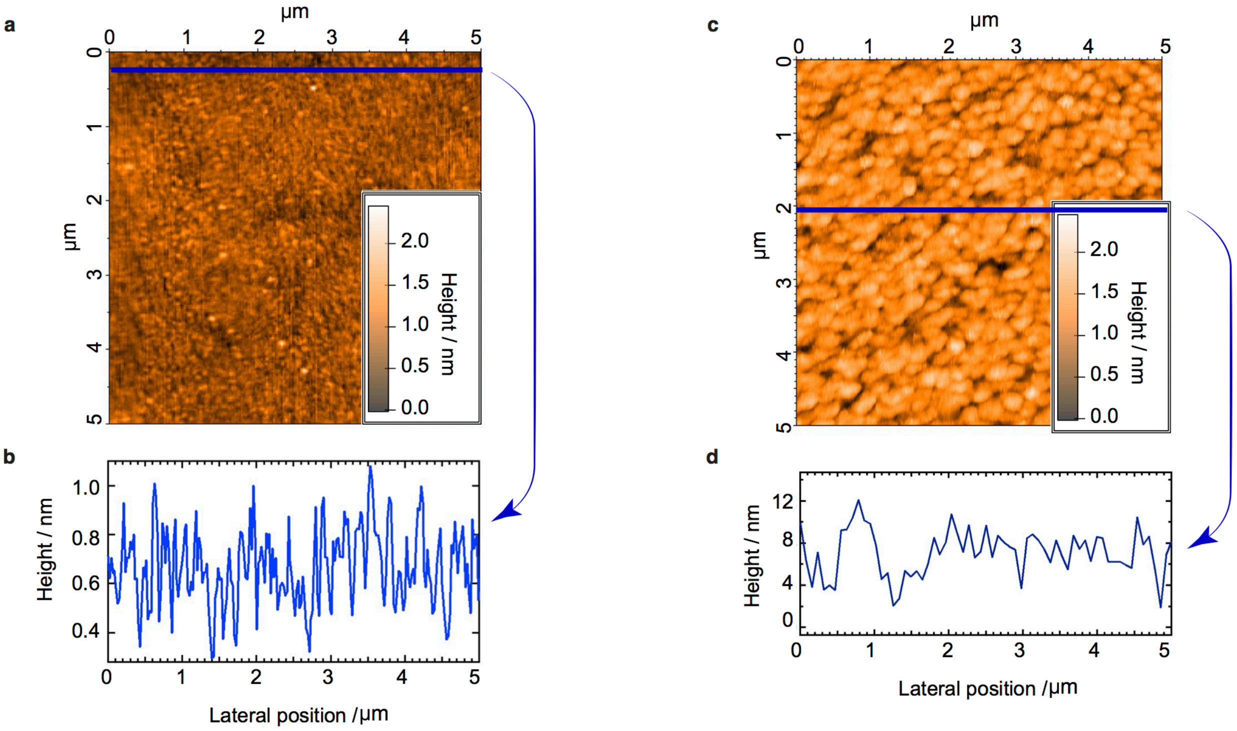

Supplementary Figure 7. AFM images. a, Fe_3O_4; **b**, surface profile of Fe_3O_4 as indicated by the blue line; **c** monolayer of $Ni(Pyipa)_2$; **d**, surface profile of $Ni(Pyipa)_2$ as indicated by the blue line.

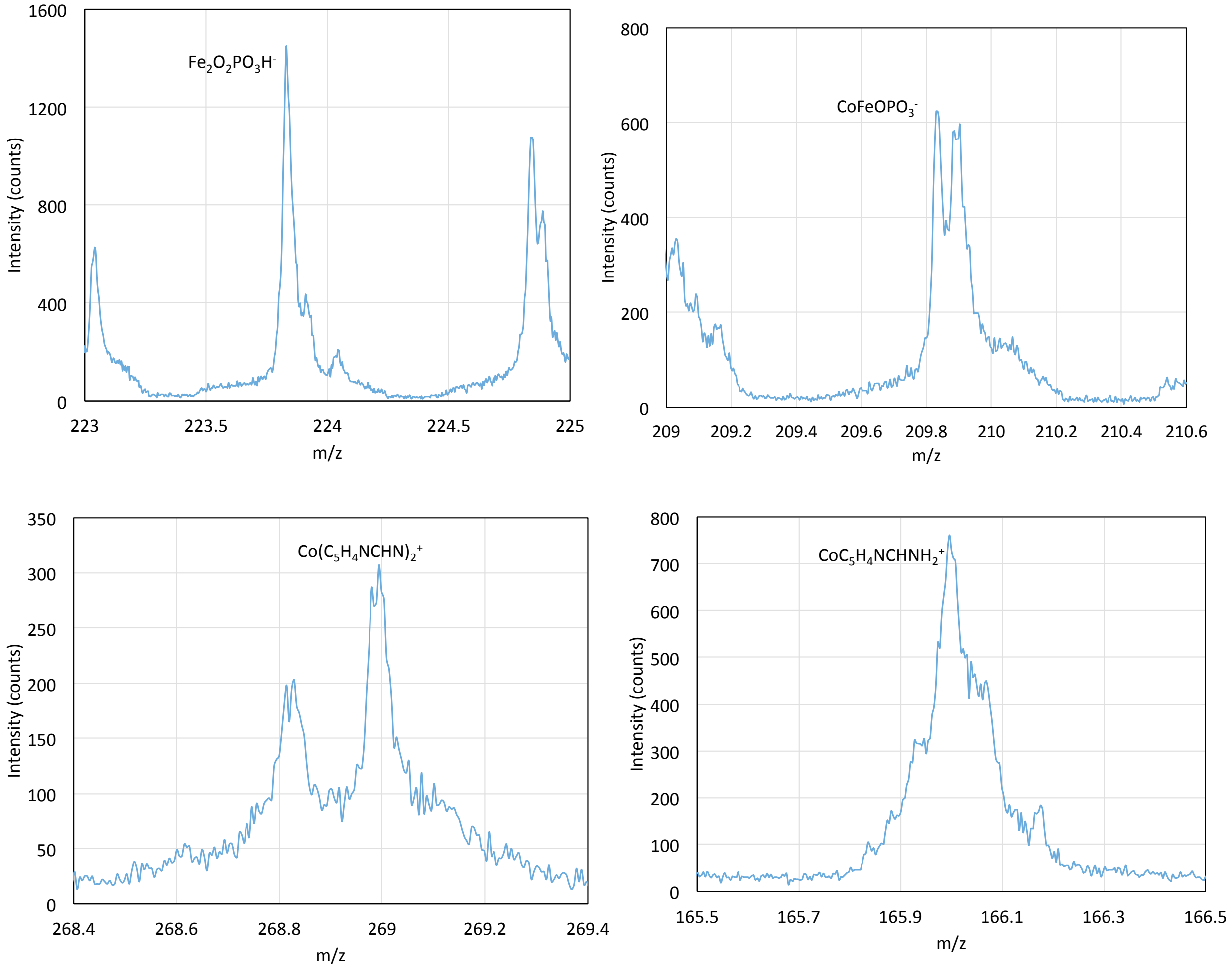

Supplementary Figure 8. Representative Tof-SIMS spectra of Co(Pyipa)$_2$ on epitaxial F_3O_4(111) for the negative (top) and positive (bottom) modes.

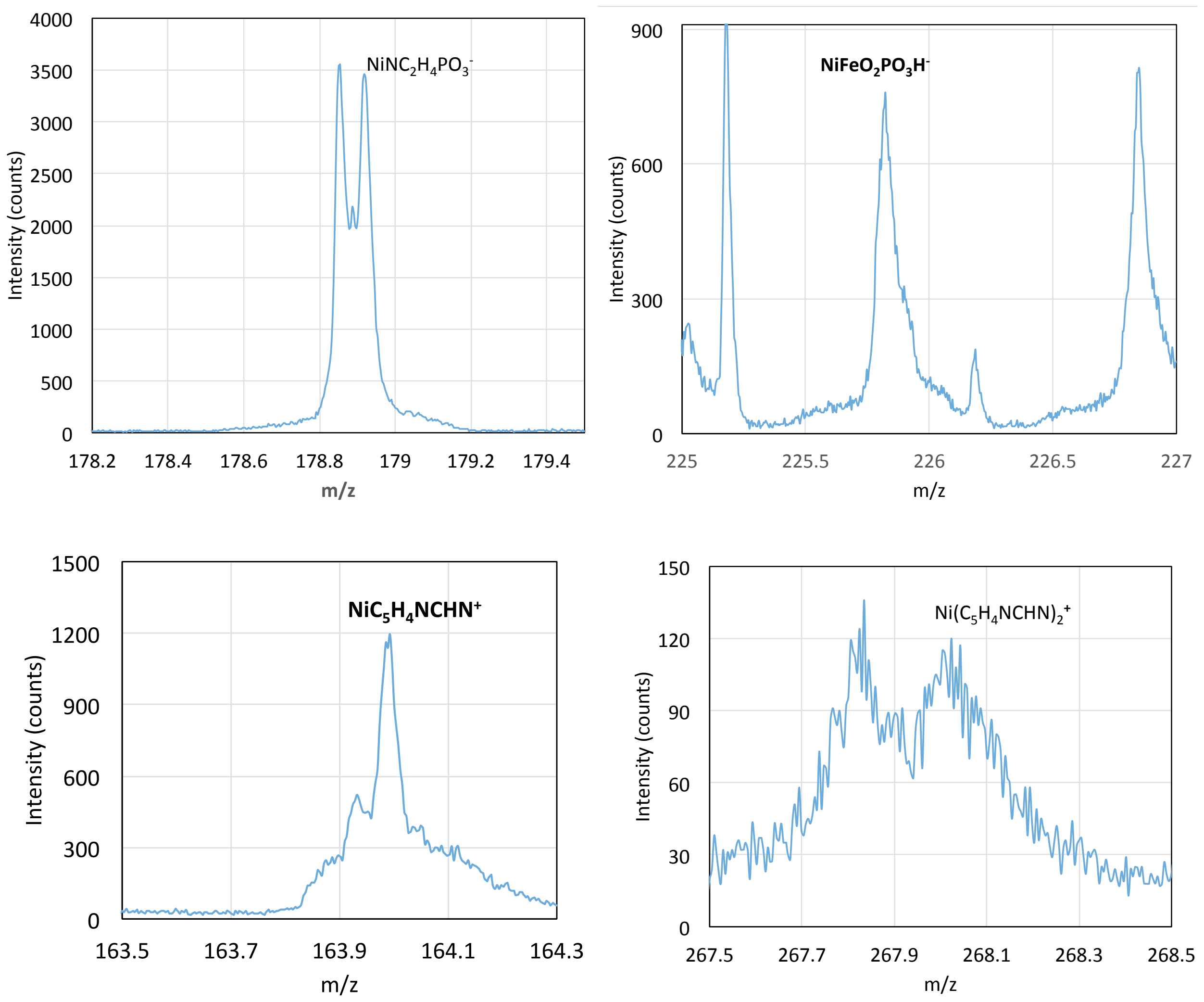

Supplementary Figure 9. Representative Tof-SIMS spectra of Ni(Pyipa)$_2$ on epitaxial F$_3$O$_4$(111) for the negative (top) and positive (bottom) modes.

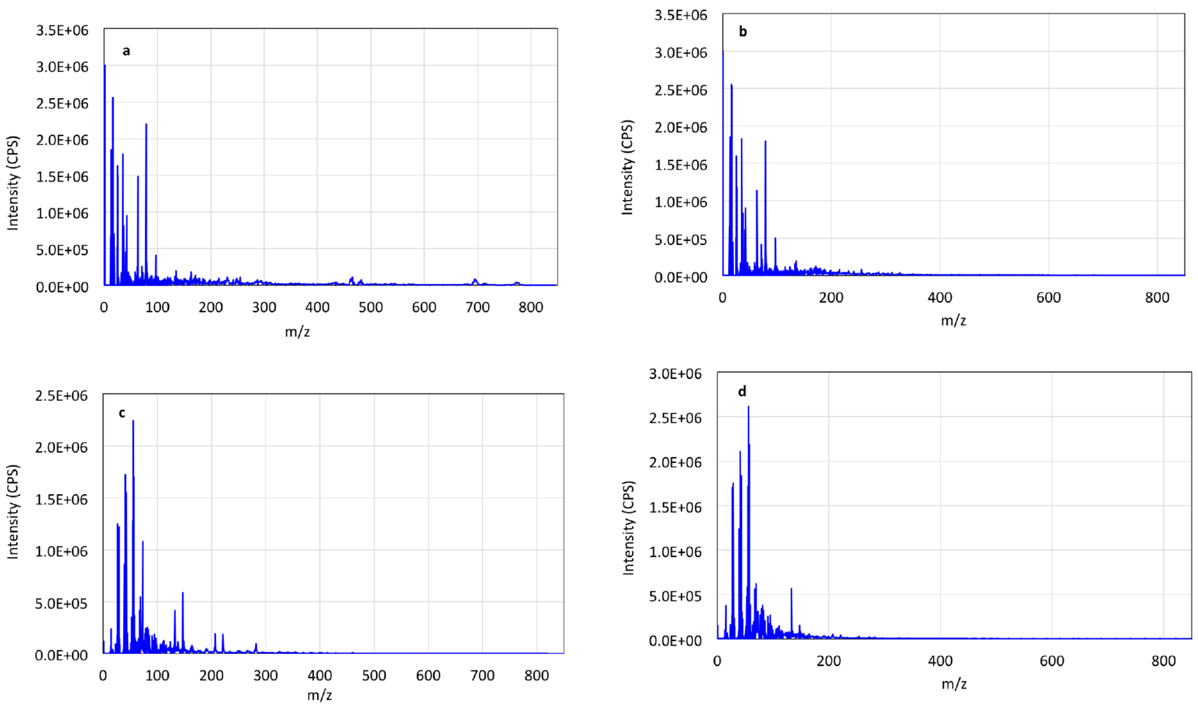

Supplementary Figure 10. Tof-SIMS spectra of Ni(Pyipa)$_2$ (a and c) and Co(Pyipa)$_2$ (b and d) on epitaxial F_3O_4(111) for the negative (top) and positive (bottom) modes.

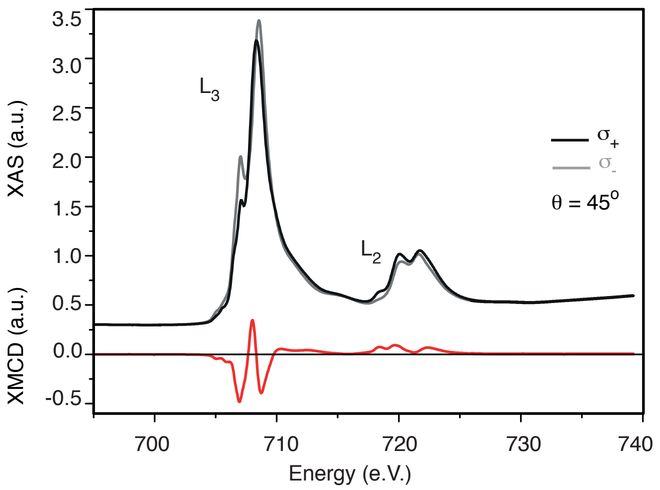

Supplementary Figure 11. XAS/XMCD of bare epitaxial Fe_3O_4(111). Iron $L_{2,3}$ edge XAS and XMCD spectra recorded at T = 2 K, and θ = 45° using left (σ_+) and right hand (σ_-) circularly polarized light in 6.5 T field.

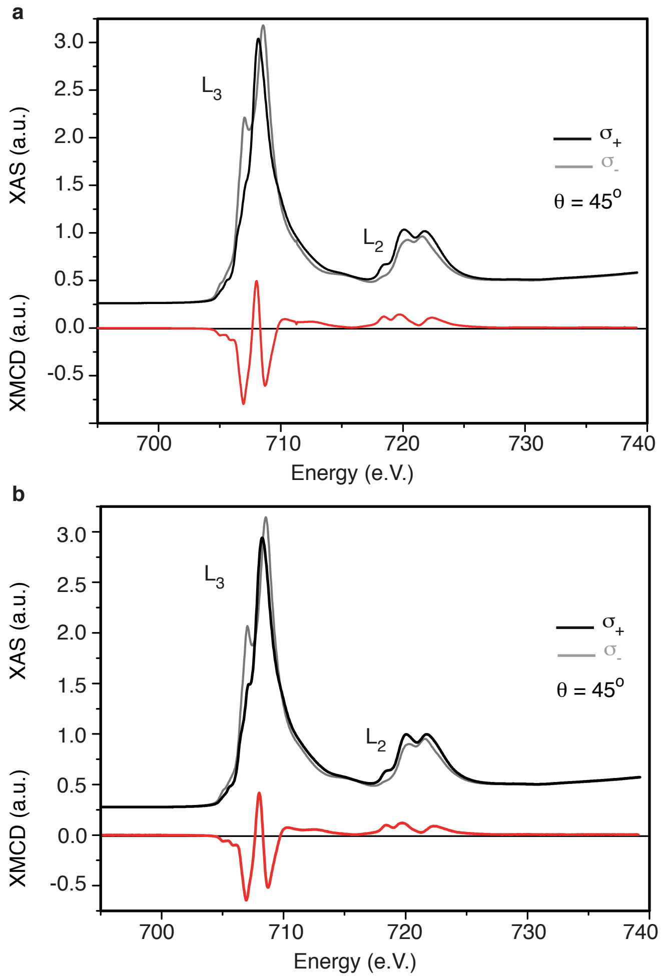

Supplementary Figure 12. XAS/XMCD of epitaxial $F_3O_4(111)$. Iron $L_{2,3}$ edge XAS and XMCD spectra recorded at T = 2 K, and θ = 45° using left (σ_+) and right hand (σ_-) circularly polarized light in 6.5 T field. **a** $Co(Pyipa)_2$ monolayer was deposited onto the Fe_3O_4; **b** $Ni(Pyipa)_2$ monolayer was deposited onto the Fe_3O_4.

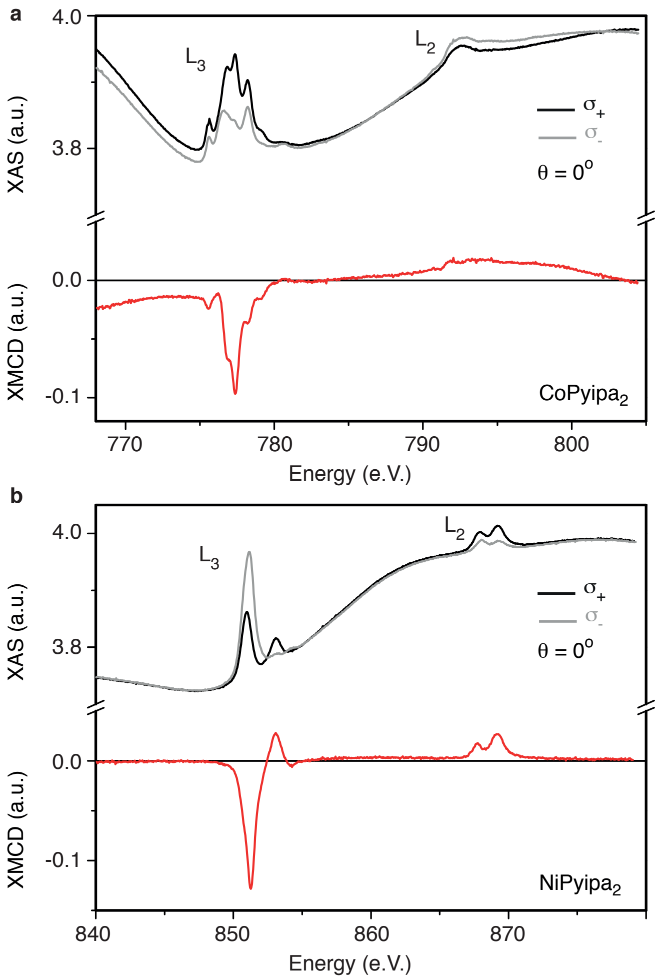

Supplementary Figure 13. XAS/XMCD of $Co(Pyipa)_2$ **and** $Ni(Pyipa)_2$**. a**, Cobalt $L_{2,3}$ edge XAS and XMCD spectra recorded at T = 2 K, and θ = 0° using left (σ_+) and right hand (σ_-) circularly polarized light in 6.5 T field. **b**, Nickel $L_{2,3}$ edge XAS and XMCD spectra recorded at T = 2 K, and θ = 0° using left (σ_+) and right hand (σ_-) circularly polarized light in 6.5 T field. Note that the non-flat background for the XMCD for Co comes from the XMCD signal of the Fe $L_{2,3}$ edges and can be removed by recording the XMCD on a pure Fe_3O_4 surface in the energy range of the Co $L_{2,3}$ edges. This step is what has been done to obtain Figures S11c and S13.

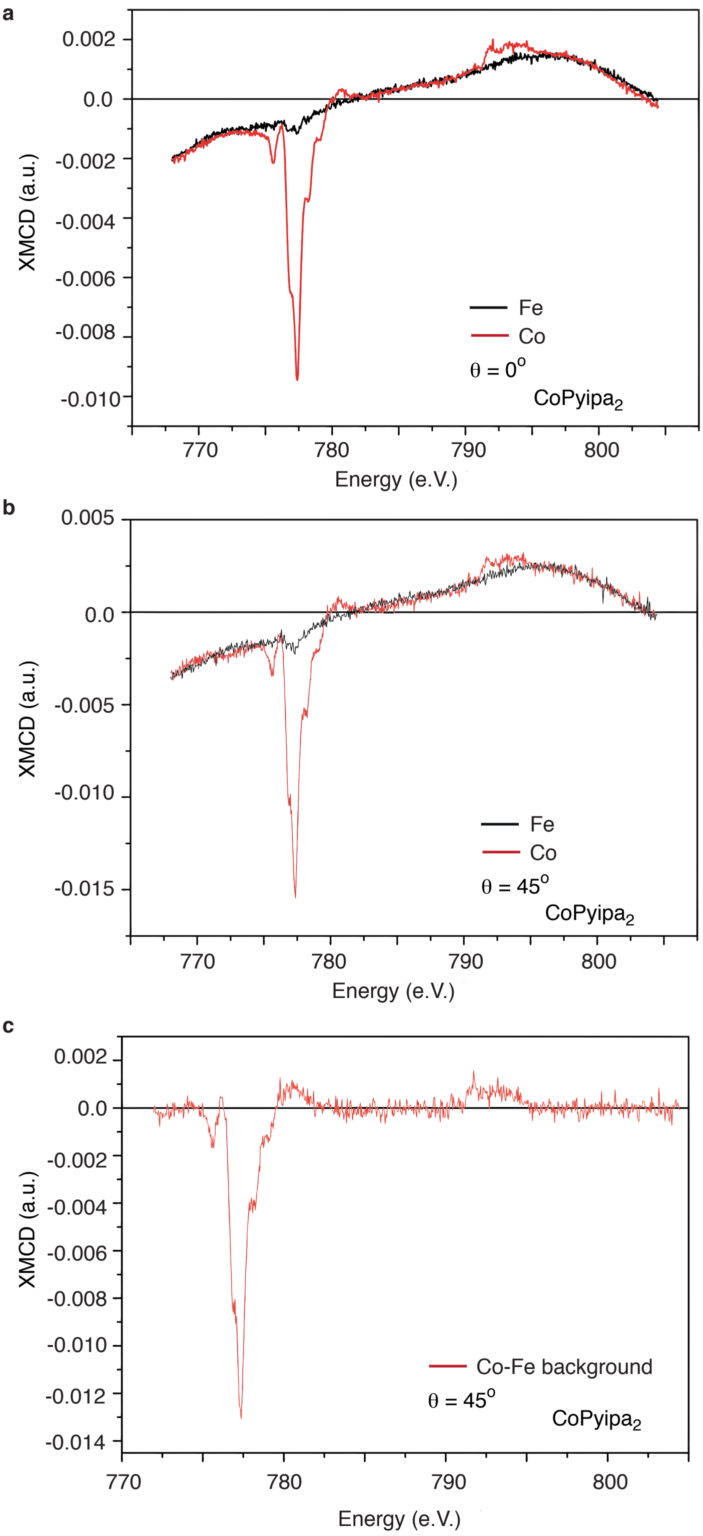

Supplementary Figure 14. XMCD of Co(Pyipa)$_2$ **and** Fe_3O_4 at the Cobalt $L_{2,3}$ edge spectra recorded at T = 2 K, θ = 0° (**a**), and θ = 45° (**b**, and **c**) using left (σ_+) and right hand (σ_-) circularly polarized light in 6.5 T field. The black line in **a** and **b** indicated the background signal arising from the Fe_3O_4 substrate. The background was subtracted to produce Figure S11c.

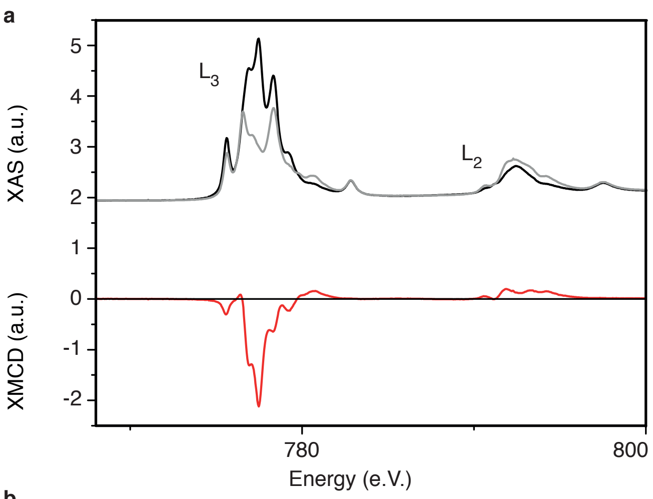

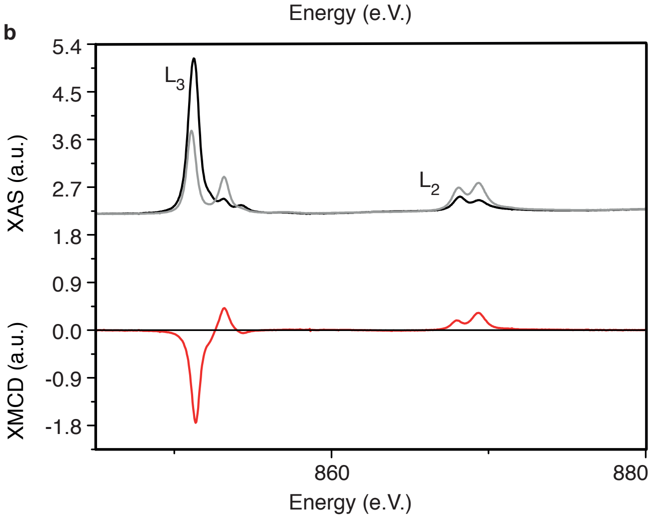

Supplementary Figure 15. XAS/XMCD of a bulk, dropped casted, layer of Co(Pyipa)$_2$ **and** Ni(Pyipa)$_2$**. a**, Cobalt $L_{2,3}$ edge XAS and XMCD spectra recorded at T = 2 K, and θ = 45° using left (σ_+) and right hand (σ_-) circularly polarized light in 6.5 T field. **b**, Nickel $L_{2,3}$ edge XAS and XMCD spectra recorded at T = 2 K, and θ = 45° using left (σ_+) and right hand (σ_-) circularly polarized light in 6.5 T field.

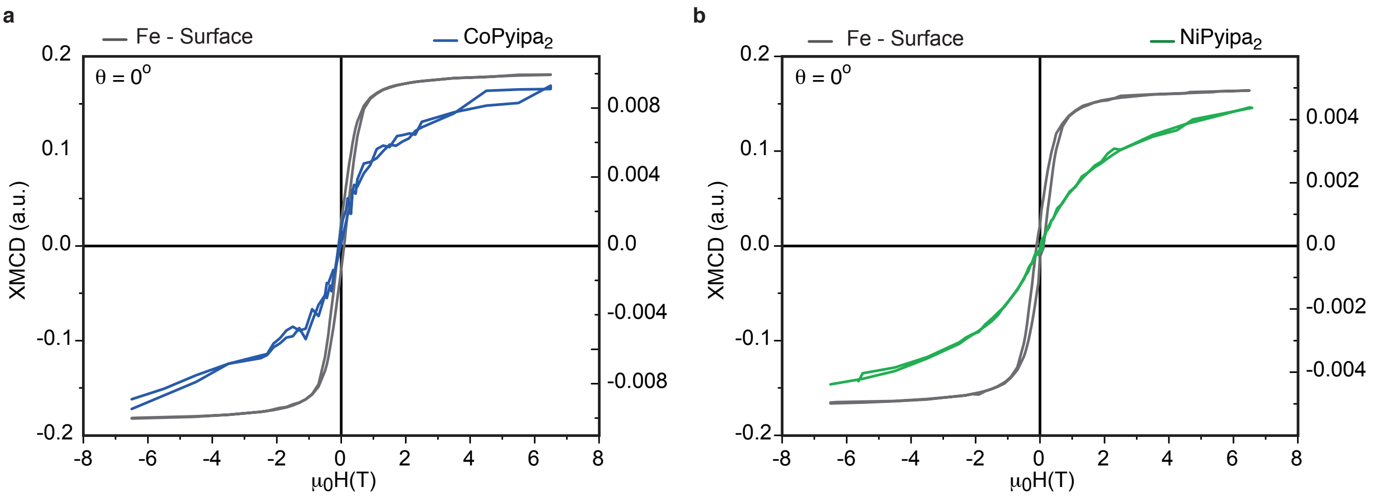

Supplementary Figure 16. Element-specific field dependence of the magnetization of the Co atoms of Co(Pyipa)$_2$, the Ni atoms of Ni(Pyipa)$_2$ and of the ferrimagnetic surface (Fe). Hysteresis curves in the ± 6 T field range (multiplied by -1) of the Co atoms (blue), Ni atoms (green) and Fe atoms (grey) obtained at the $L_{2,3}$ edges XMCD maxima at T = 2 K, and θ = 0°. (Monochromatized X-rays are set at the energy of the maximum absolute value of the XMCD signal (i.e. hν = 777.5 eV for Co, hν = 851 eV for Ni, and hν = 707 eV for Fe) then the external magnetic field is switched step by step from +6.5 T down to -6.5 T and back to +6.5 T. At each step the magnetic field is switched from left to right circular polarization to yield the element specific magnetization curves.

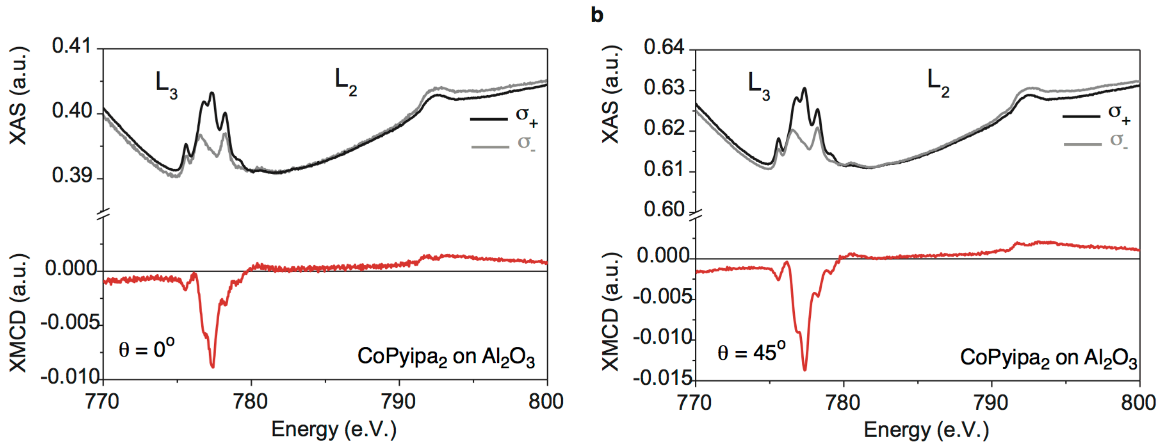

Supplementary Figure 17. XAS/XMCD of Co(Pyipa)$_2$ deposited onto an Al_2O_3 terminated Fe_3O_4. Cobalt $L_{2,3}$ edge XAS and XMCD spectra recorded at T = 2 K, θ = 0° (red line), and θ = 45° (black line) using left (σ_+) and right hand (σ_-) circularly polarized light in 6.5 T field.

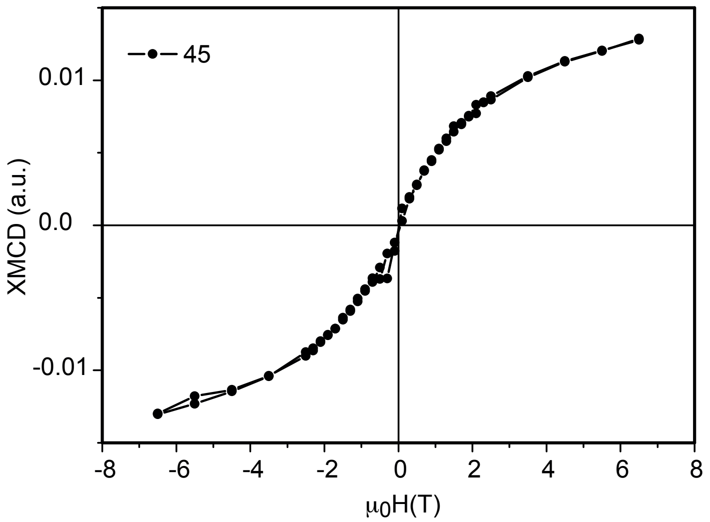

Supplementary Figure 18. Element-specific field dependence of the magnetization of the Co atoms of Co(Pyipa)$_2$ on the ferrimagnetic surface (Fe) separated by an Al_2O_3 insulating layer. Hysteresis curves (multiplied by -1) of the Co atoms obtained at the $L_{2,3}$ edges XMCD maxima at T = 2 K, θ = 45°. (Monochromatized X-rays are set at the energy of the maximum absolute value of the XMCD signal (i.e. hν = 777.5 eV for Co) then the external magnetic field is switched step by step from +6.5 T down to -6.5 T and back to +6.5 T. At each step the magnetic field is switched from left to right circular polarization to yield the element specific magnetization curves.

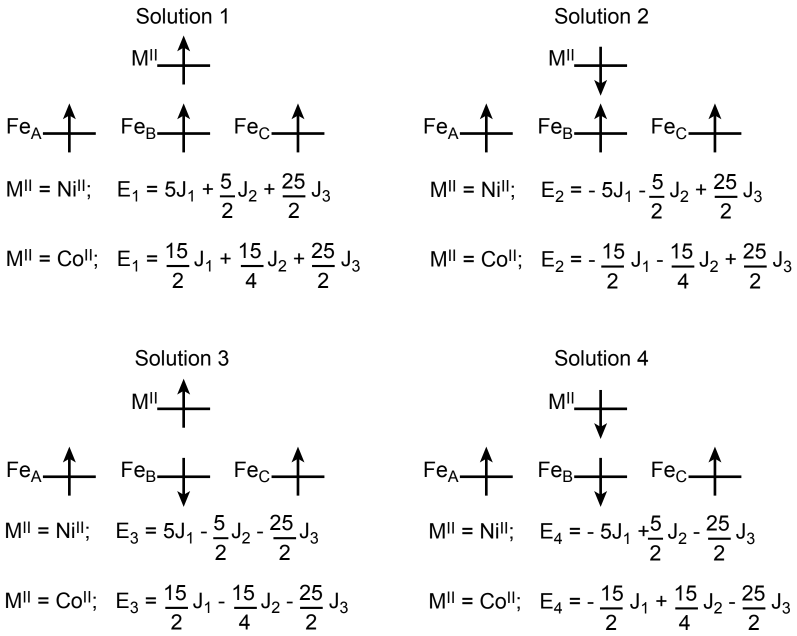

Supplementary Figure 19. DFT broken symmetry solutions and their analytical energies as functions of the magnetic exchange coupling parameters J_1, J_2 and J_3. Solutions 1 and 2 correspond to the situation when the surface Fe^{III} ions are *ferromagnetically* coupled and the M^{II} ions are ferro- and antiferromagnetically coupled to them, respectively; solutions 3 and 4 correspond to the situation when the surface Fe^{III} ions are *antiferromagnetically* coupled and the M^{II} ions are ferro- and antiferromagnetically coupled to them, respectively. Although solutions 1 and 2 (ferromagnetic coupling between Fe ions of the surface) are lower in energy than solutions 3 and 4 (antiferromagnetic coupling between Fe ions of the surface), the coupling between the complexes and the substrate is independent from the nature of the coupling within the substrate because equations $E_1 – E_2$ and $E_3 – E_4$ are a function of J_1 and J_2 only

Supplementary Table 1. Crystallographic data for complex Co(Pyipa)$_2$ and Ni(Pyipa)$_2$.

**Compound**	**Co(Pyipa)$_2$**	**Ni(Pyipa)$_2$**
Formula	$C_{16}CoH_{20}N_4O_6P_2$,	$C_{16}H_{20}N_4NiO_6P_2$,
fw	485.23	484.99
Crystal size / mm^3	0.11 x 0.07 x 0.04	0.24 x 0.06 x 0.01
Crystal system	monoclinic	monoclinic
Space group	*C 2/c*	*C 2/c*
a, Å	26.3780(13)	25.9071(8)
b, Å	9.0590(4)	9.1614(3)
c, Å	16.5517(7)	16.4713(4)
α, °	90	90
β, °	102.2860(10)	100.9560(10)
γ, °	90	90
Cell volume, Å^3	3864.6(3)	3838.1(2)
Z	8	8
T, K	100(1)	100(1)
F_{000}	1992	2000
μ / mm^{-1}	1.097	1.221
θ range / °	1.58 – 30.51	1.60 – 30.51
Refl. collected	29 403	29 473
Refl. unique	5 844	5 540
R_{int}	0.0616	0.0340
GOF	1.031	1.036
Refl. obs. ($I>2\sigma(I)$)	3 926	4 321
Parameters	676	266
wR_2 (all data)	0.1299	0.1401
R value ($I>2\sigma(I)$)	0.0524	0.0540
Largest diff. peak and hole	-0.967; 1.996	-1.268 ; 1.642

Supplementary Table 2. XPS peak ratio of the fresh and 2-day incubated samples.

**Ni samples**	**%Ni**	**%N**	**%P**
fresh	14	57	29
2-days incubation	14	54	32
**Co samples**	**%Co**	**%N**	**%P**
fresh	14	54	32
2-days incubation	14	52	34
theoretical	1	4	2

Supplementary Table 3. Ions detected by Tof-SIMs for Co(Pyipa)$_2$. Important peaks are highlighted in yellow.

Co system	detected mass (m/z)	theoretical mass (m/z)
Co^-	58.93	58.933
$CoPO_3^-$	137.90	137.892
$FeOPO_3^-$	150.89	150.888
$CoPO_3H^-$	138.90	138.900
$CoPO_3FeO^-$	209.83	209.822
$Co(PO_3)_2FeO^-$	288.78	288.780
$Co(PO_3)_2FeO_2^-$	304.76	304.775
$N(CH_2)_2CoPO_3H^-$	180,93 (sh)	180.934
$N(CH_2)_2CoPO_3^-$	179,90 (sh)	179.926
Co^+	58,93 (sh)	58.933
CoH^+	59.94	59.941
$CoC_5H_4NCHN^+$	163,98 (sh)	163.978
$CoC_5H_4NCHNH^+$	164,99 (weak)	164.986
$CoC_5H_4NCHNH_2^+$	165.99	165.994
$CoC_5H_4NCHNCH_2^+$	178 (weak)	177.994
$CoC_5H_4NCHNCH_3^+$	179 (weak)	179.002
$CoC_5H_4NCHNC_2H_5^+$	193,02 (weak)	193.017
$Co(C_5H_4NCHN)_2^+$	268.99	269.024

Supplementary Table 4. Ions detected by Tof-SIMs for Ni(Pyipa)$_2$. Important peaks are highlighted in yellow.

Ni system	detected mass (m/z)	theoretical mass (m/z)
Ni^-	57.94	57.935
NiH^-	58.94	58.943
$NiFeOPO_3H^-$	209.83	209.831
$NiFeO_2PO_3H^-$	225.83	225.826
$NiNC_2H_4PO_3^-$	178.92	178.928
Ni^+	57.94	57.935
NiH^+	58.94	58.943
$NiFe^+$	113.87	113.870
$NiFeO^+$	129.87	129.865
$NiC_5H_4NCHN^+$	162.99	162.981
$NiC_5H_4NCHNH^+$	163.99	163.988
$NiC_5H_4NCHNCH_2^+$	176.99	176.996
$NiC_5H_4NCHNCH_3^+$	178.00	178.004
$NiC_5H_4NCHNC_2H_5^+$	192.02	192.020
$Ni(C_5H_4NCHN)_2^+$	268.02	268.026

Supplementary Table 5. Energies in a.u. of the four broken symmetry solutions calculated at the B3LYP level and depicted in Figure S16 for the Ni and Co complexes.

M/E(a.u.)	E1	E2	E3	E4
Ni	-8187.1889526	-8187.1897272	-8187.1843507	-8187.1852164
Co	-8061.646066	-8061.640701	-8061.639038	-8061.637022

Supplementary Discussion

***Ab initio* calculations of the complexes**. *Ab initio* calculations using the spin-orbit state interaction (SOSI) method, which treats the spin-orbit coupling (SOC)[1,2] in the wave function theory (WFT)- based framework (implemented in the MOLCAS code[3]) permits the calculation of the ZFS parameters from first principles and the determination of the orientation of the main axes of the anisotropy tensor according to a well-established method, which is known to provide accurate results.[4-10] The calculations, in very good agreement with experimental data, lead to D_{Ni} = -3.4 cm^{-1} and E_{Ni} = 0.7 cm^{-1}, which corresponds to a weak separation between the ground M_S = ±1 (not degenerate because $E \neq 0$ for a non Kramers sub-levels) and the excited M_S = 0 of 3.4 cm^{-1}. The easy axis of magnetization was found to be perpendicular to the C_2 symmetry axis of the molecule and makes an angle of 10° with the N_{amine}-Ni-N_{amine} direction. For $Co(Pyipa)_2$, the calculation gives D_{Co} = + 32.3 cm^{-1} and E_{Co} = 4.1 cm^{-1}, which corresponds to a large energy separation between the ground M_S = ±1/2 and the excited M_S = ± 3/2 sub-levels of 64.6 cm^{-1} (= 2|D|).

XPS analysis. For the 2-day incubated samples, XPS fitting was performed using CasaXPS and IgorPro Wavemetrics. Photoemitted electron data spectra were decomposed using mixed Lorenzian and Gaussian (1/3;2/3) peaks for carbon, phosphorus and nitrogen. In the case of metal, e.g. cobalt and nickel, the peaks are composed of Gaussian and Lorenzian peaks. The energetic splitting for 2P 1/2 and 2P 3/2 for the phosphorus element was fixed at 0.7 eV, and the intensity set at 1/3 to 2/3 respectively. The atomic ratio was determined using the surface area of each peak for each element taking in consideration the Scofield factors. For the as-dissolved samples, the areas of the peaks were measured after background correction of Shirley type,[11] and then corrected using Scofield parameters (Ni-2p3/2 : 14.610 ; Co-2p3/2 : 12.620 ; N-1s : 1.80 et P-2p : 1.192). These procedures allow quantification of the relative percentages of the three elements M (Ni or Co), N and P for the four samples shown in supplementary Table 2.

Density Functional Theory – Geometrical optimization of the complexes on the surface. In our calculations, we have considered an 8 ML slab of 4 x 4 unit cells of Fe_3O_4 oriented along the (111) direction and two *k*-points. We have set the molecule in three different initial configurations, namely a physisorbed configuration and two chemisorbed configurations, considering the anchoring of the molecule with two

equivalent or non-equivalent oxygen atoms of the surface. Then, we have proceeded to a DFT molecular dynamic simulation at room temperature to reproduce the experimental conditions of molecular deposition on the surface, followed by a structural optimization at 0K to determine the final configuration on the surface. This well-established procedure[12] gives us a stable structure in the three configurations, as well as the molecular adsorption energies:

Physisorption: -2.51 eV/molecule

Chemisorption (non equivalent O): -8.14 eV/molecule

Chemisorption (equivalent O): -10.11 eV/molecule

The latter configuration seems to be the most stable, defining the molecular orientation observed in the experiments and more importantly the site where the molecules are linked to the Fe surface atoms.

Sum rule calculations. For Co^{II} (in a monolayer of $Co(Pyipa)_2$) the number of hole was taken to be $n_h = 3$, which results, at θ = 0°, in an orbit magnetic moment $M_L = -\mu_B \langle L_Z \rangle = 0.39 \pm 0.03\ \mu_B$ and a spin magnetic moment $M_S = -g_0\mu_B \langle S_Z \rangle = 1.19 \pm 0.08\ \mu_B$, corresponding to a total magnetic moment $M = M_L + M_S = 1.58 \pm 0.11\ \mu_B$. For Co^{II} (in a monolayer of $Co(Pyipa)_2$) the number of hole was taken, again, to be $n_h = 3$, which results, at θ = 45°, in an orbit magnetic moment $M_L = -\mu_B \langle L_Z \rangle = 0.44 \pm 0.03\ \mu_B$ and a spin magnetic moment $M_S = -g_0\mu_B \langle S_Z \rangle = 1.39 \pm 0.08\ \mu_B$, corresponding to a total magnetic moment $M = M_L + M_S = 1.83 \pm 0.11\ \mu_B$. For Ni^{II} (in a monolayer of $Ni(Pyipa)_2$) the number of hole was taken, again, to be $n_h = 2$, which results, at θ = 45°, in an orbit magnetic moment $M_L = -\mu_B \langle L_Z \rangle = 0.35 \pm 0.02\ \mu_B$ and a spin magnetic moment $M_S = -g_0\mu_B \langle S_Z \rangle = 1.39 \pm 0.12\ \mu_B$, corresponding to a total magnetic moment $M = M_L + M_S = 1.74 \pm 0.14\ \mu_B$ (the error bars are estimated to 10 % and stem mainly from the normalization procedure and the background subtraction). For Ni^{II} (in a monolayer of $Ni(Pyipa)_2$) the number of hole was taken to be $n_h = 2$, which results, at θ = 45°, in an orbit magnetic moment $M_L = -\mu_B \langle L_Z \rangle = 0.34 \pm 0.02\ \mu_B$ and a spin magnetic moment $M_S = -g_0\mu_B \langle S_Z \rangle = 1.50 \pm 0.12\ \mu_B$, corresponding to a total magnetic moment $M = M_L + M_S = 1.84 \pm 0.14\ \mu_B$ (the error bars are estimated to 10 % and stem mainly from the normalization procedure and the background subtraction). The values for both Co and Ni in a thick monolayer are comparable to those found in the monolayer at θ = 45°. For Co^{II} (in a monolayer of $Co(Pyipa)_2$ deposited onto Al_2O_3) the number of hole was taken to be $n_h = 3$, which

results, at θ = 0°, in an orbit magnetic moment $M_L = -\mu_B \langle L_Z \rangle = 0.60 \pm 0.03\ \mu_B$ and a spin magnetic moment $M_S = -g_0\mu_B \langle S_Z \rangle = 1.52 \pm 0.08\ \mu_B$, corresponding to a total magnetic moment $M = M_L + M_S = 2.12 \pm 0.14\ \mu_B$ (the error bars are estimated to 10 % and stem mainly from the normalization procedure and the background subtraction). For Co^{II} (in a monolayer of $Co(Pyipa)_2$ deposited onto Al_2O_3) the number of hole was taken, again, to be $n_h = 3$, which results, at θ = 45°, in an orbit magnetic moment $M_L = -\mu_B \langle L_Z \rangle = 1.45 \pm 0.03\ \mu_B$ and a spin magnetic moment $M_S = -g_0\mu_B \langle S_Z \rangle = 1.39 \pm 0.08\ \mu_B$, corresponding to a total magnetic moment $M = M_L + M_S = 2.84 \pm 0.14\ \mu_B$ (the error bars are estimated to 10 % and stem mainly from the normalization procedure and the background subtraction).

Density Functional Theory – calculation of the magnetic coupling between the molecules the surface. These calculations have been performed using the B3LYP functional of the GAUSSIAN 09 package.[13,14] First, the structure of each complex anchored to the surface was optimized. The effect of the surface onto the complex was modeled as follows: a piece containing three Fe^{III} centers and the coordinated O atoms (see Figure 5) was cut into the structure that was predetermined by the molecular dynamics technique. The external oxo anions were protonated. It is important to note that even if the surface model is approximated, it is exactly the same for both complexes. The structure of the model surface was frozen and the structure of the whole complex was optimized for the $M_s = 18/2$ ($Co(Pyipa)_2$) or $M_s = 17/2$ ($Ni(Pyipa)_2$) solution corresponding to a ferromagnetic coupling between the three Fe^{III} and the Co^{II} or Ni^{II} metal centers. Very small spin contamination is observed: for $Co(Pyipa)_2$, $S = 9.03$, compared to an expected value of $S = 9.00$ without spin contamination; for $Ni(Pyipa)_2$, $S = 8.52$ *vs* an expected value of $S = 8.50$. For both complexes, the spin density of the central the Fe^{III} centers are almost the same and independent of the M_s values. A substantial spin density delocalization toward the O atoms bound to the Fe centers is observed in both cases (as usual in metal oxides).[15-19] The Co^{II} and Ni^{II} ions have spin densities of 1.33 and 0.86, respectively. Unexpectedly, the coordination sphere of $Ni(Pyipa)_2$ is slightly smaller than that of $Co(Pyipa)_2$ as can be seen from the differences in bond distances: Ni-N distances are between 2.03Å and 2.15Å (average 2.10Å); Ni-O distances are 2.03Å and 2.08Å; Co-N distances are between 2.05Å and 2.19Å (average 2.14Å); Co-O distances are 2.09Å and 2.24Å. The distance

from the metal center to the closest Fe^{III} ion is shorter by 0.5 Å for Ni $(Pyipa)_2$ than that of Co $(Pyipa)_2$ (4.27Å *vs.* 4.77Å).

Second, the solution corresponding to a ferromagnetic coupling between the Fe^{III} centers and an antiferromagnetic coupling with the Co^{II} and Ni^{II} ions was computed (M_s = 12/2 and M_s = 13/2 for Co- and Ni$(Pyipa)_2$, respectively). It should be noted that the extraction of the magnetic couplings J_1 and J_2 is independent of the nature of the surface couplings, since the equations E_1-E_2 and E_3-E_4 which have been used are functions of J_1 and J_2 only. As expected, the spin contamination of these solutions is larger but it remains small: S = 6.28 (exp. 6.00) for Co$(Pyipa)_2$; S = 6.67 (exp. 6.50) for Ni$(Pyipa)_2$. The spin densities on the iron centers are almost the same as in the high-spin solution (variations of less than 0.02); that of Co^{II} and Ni^{II} are -1.40 and -0.87, respectively.

Supplementary References

1 Malmqvist, P. A., Roos, B. O. & Schimmelpfennig, B. The restricted active space (RAS) state interaction approach with spin-orbit coupling. *Chem. Phys. Lett.* **357**, 230-240, (2002).

2 Roos, B. O. & Malmqvist, P.-A. Relativistic quantum chemistry: the multiconfigurational approach. *Phys. Chem. Chem. Phys.* **6**, 2919-2927, (2004).

3 Karlstroem, G. *et al.* MOLCAS: a program package for computational chemistry. *Comput. Mater. Sci.* **28**, 222-239, (2003).

4 Ruamps, R. *et al.* Giant Ising-Type Magnetic Anisotropy in Trigonal Bipyramidal Ni(II) Complexes: Experiment and Theory. *J. Am. Chem. Soc.* **135**, 3017-3026, (2013).

5 Maurice, R., Graaf, C. d. & Guihéry, N. Theoretical determination of spin Hamiltonians with isotropic and anisotropic magnetic interactions in transition metal and lanthanide complexes. *Phys. Chem. Chem. Phys.* **15**, 18784, (2013).

6 Ruamps, R. *et al.* Origin of the Magnetic Anisotropy in Heptacoordinate Ni^{II} and Co^{II} Complexes. *Chem. -Eur. J.* **19**, 950-956, (2012).

7 Batchelor, L. J. *et al.* Pentanuclear cyanide-bridged complexes based on highly anisotropic Co(II) seven-coordinate building blocks: synthesis, structure, and magnetic behavior. *Inorg. Chem.* **50**, 12045-12052, (2011).

8 Maurice, R., Guihéry, N., Bastardis, R. & Graaf, C. d. Rigorous Extraction of the Anisotropic Multispin Hamiltonian in Bimetallic Complexes from the Exact Electronic Hamiltonian. *J. Chem. Theory Comput.* **6**, 55-65, (2010).

9 Maurice, R., de Graaf, C. & Guihéry, N. Magnetic anisotropy in binuclear complexes in the weak-exchange limit: From the multispin to the giant-spin Hamiltonian. *Phys. Rev. B* **81**, 214427, (2010).

10 Maurice, R. *et al.* Universal Theoretical Approach to Extract Anisotropic Spin Hamiltonians. *J. Chem. Theory Comput.* **5**, 2977-2984, (2009).

11 Végh, J. The analytical form of the Shirley-type background. *J. Electron Spectrosc. Relat. Phenom.* **46**, 411-417, (1988).

12 Schull, G., Dappe, Y. J., González, C. s., Bulou, H. & Berndt, R. Charge Injection through Single and Double Carbon Bonds. *Nano Letters* **11**, 3142-3146, (2011).

13 Gaussian 03 (Revision B.05) (Gaussian, Inc., Pittsburgh, PA, 2003).

14 Gaussian 09 (Revision C.01) (Gaussian, Inc., Pittsburgh, PA, 2003).

15 Gellé, A., Munzarová, M. L., Lepetit, M.-B. & Illas, F. Role of dynamical polarization of the ligand-to-metal charge transfer excitations in *ab initio* determination of effective exchange parameters. *Phys. Rev. B* **68**, 125103, (2003).

16 Labèguerie, P. *et al.* Is it possible to determine rigorous magnetic Hamiltonians in spin s=1 systems from density functional theory calculations? *J. Chem. Phys.* **129**, 154110, (2008).

17 Calzado, C. J. On the Controversial Fitting of Susceptibility Curves of Ferromagnetic CuII Cubanes: Insights from Theoretical Calculations. *Chem.-Eur. J.* **19**, 1254-1261, (2013).

18 Malrieu, J. P., Caballol, R., Calzado, C. J., de Graaf, C. & Guihéry, N. Magnetic Interactions in Molecules and Highly Correlated Materials: Physical Content, Analytical Derivation, and Rigorous Extraction of Magnetic Hamiltonians. *Chem. Rev.* **114**, 429-492, (2014).

19 Noh, J., Osman, O. I., Aziz, S. G., Winget, P. & Bredas, J.-L. A density functional theory investigation of the electronic structure and spin moments of magnetite. *Sci. Tech. Adv. Mater.* **15**, 044202, (2014).
